# Using virtual patient cohorts to uncover immune response differences in cancer and immunosuppressed COVID-19 patients

**DOI:** 10.1371/journal.pcbi.1013170

**Published:** 2025-06-09

**Authors:** Sonia T. Gazeau, Xiaoyan Deng, Elsa Brunet-Ratnasingham, Daniel E. Kaufmann, Catherine Larochelle, Penelope A. Morel, Jane M. Heffernan, Courtney L. Davis, Amber M. Smith, Adrianne L. Jenner, Morgan Craig

**Affiliations:** 1 Sainte-Justine University Hospital Research Centre, Montréal, Québec, Canada; 2 Department of Mathematics and Statistics, Université de Montréal, Montréal, Québec, Canada; 3 University of California, San Francisco, California, United States of America; 4 Research Centre of the Centre Hospitalier de l’Université de Montréal (CRCHUM), Montréal, Québec, Canada; 5 Division of Infectious Diseases, Department of Medicine, Lausanne University Hospital (CHUV) and Université de Lausanne, Lausanne, Switzerland; 6 Department of Immunology, University of Pittsburgh, Pittsburgh, Pennsylvania, United States of America; 7 Centre for Disease Modelling, Department of Mathematics & Statistics, York University, Toronto, Ontario, Canada; 8 Natural Science Division, Pepperdine University, Malibu, California, United States of America; 9 Department of Pediatrics, University of Tennessee Health Science Center, Memphis, Tennessee, United States of America; 10 School of Mathematical Sciences, Queensland University of Technology, Brisbane, Queensland, Australia; Los Alamos National Laboratory, UNITED STATES OF AMERICA

## Abstract

The COVID-19 pandemic caused by the severe acute respiratory syndrome coronavirus-2 (SARS-CoV-2) resulted in millions of deaths globally. Adults with immunosuppression (e.g., solid organ transplant recipients) and those undergoing active cancer treatments experience worse infections and more severe COVID-19. It is difficult to conduct clinical studies in these populations, resulting in a restricted amount of data that can be used to relate mechanisms of immune dysfunction to COVID-19 outcomes in these vulnerable groups. To study immune dynamics after infection with SARS-CoV-2 and to investigate drivers of COVID-19 severity in individuals with cancer and immunosuppression, we adapted our mathematical model of the immune response during COVID-19 and generated virtual patient cohorts of cancer and immunosuppressed patients. The cohorts of plausible patients recapitulated available longitudinal clinical data collected from patients in Montréal, Canada area hospitals. Our model predicted that both cancer and immunosuppressed virtual patients with severe COVID-19 had decreased CD8 + T cells, elevated interleukin-6 concentrations, and delayed type I interferon peaks compared to those with mild COVID-19 outcomes. Additionally, our results suggest that cancer patients experience higher viral loads (however, with no direct relation with severity), likely because of decreased initial neutrophil counts (i.e., neutropenia), a frequent toxic side effect of anti-cancer therapy. Furthermore, severe cancer and immunosuppressed virtual patients suffered a high degree of tissue damage associated with elevated neutrophils. Lastly, parameter values associated with monocyte recruitment by infected cells were found to be elevated in severe cancer and immunosuppressed patients with respect to the COVID-19 reference group. Together, our study highlights that dysfunctions in type I interferon and CD8 + T cells are key drivers of immune dysregulation in COVID-19, particularly in cancer patients and immunosuppressed individuals.

## Authors summary

Individuals with active cancer disease and/or immunosuppression due to organ or stem cell transplantation often suffer from more severe COVID-19. These patients typically have CD8^+^ T cell deficiency and increased IL-6 concentrations, which is thought to weaken their immune response and increase their vulnerability to severe infections. To better predict COVID-19 severity in patients with cancer or immunosuppression, we paired a mechanistic mathematical model of the immune response to SARS-CoV-2 with clinical data from these patients. Comparing their predicted model trajectories to those from a reference COVID-19 virtual patient cohort suggested that cancer patients tend to have higher viral load peaks compared to other patients, which our model predicted to be due to pre-existing neutropenia. In addition, both immunosuppressed and cancer virtual patients exhibited increased monocyte recruitment and overall more lung tissue damage than those in the reference group. The main severity indicators in all virtual patients were found to be a delayed type I IFN peak and depressed CD8 + T cell concentrations, particularly in cancer and immunosuppressed patients. However, our model predicted that neutrophils were substantially increased only in severe cancer and immunosuppressed patients. Together, this study uses virtual patient cohorts in complement to experimental and clinical studies to better understand the immunological dysregulation leading to severe COVID-19 in vulnerable patient groups.

## Introduction

The COVID-19 pandemic caused by the severe acute respiratory syndrome coronavirus-2 (SARS-CoV-2) caused more than 7 million deaths globally as of July 2024 [1]. COVID-19 results in heterogeneous immune responses and outcomes, where some individuals experience no or very few symptoms while others become hyperinflamed and may need supportive oxygen or succumb to the infection. The risk of severe complications to SARS-CoV-2 infection is increased for individuals with weakened or suppressed immune responses [2]. Thus, it is critically important to study immuno-infection dynamics, especially in vulnerable groups (e.g., immunocompromised individuals [3–5] such as patients receiving immunosuppressants after organ transplantation, cancer patients [6–8], older adults [9,10]) whose immune systems may not adequately protect against the virus and who may have imperfect vaccine-induced immune responses [11].

Cancer patients tend to have weaker responses to viral infections [7], mostly due to impaired responses of type I interferon (IFN) that are typical of cancers [12] and diverse immune cell dysfunctions that are frequent adverse effects of oncologic treatments. COVID-19 mortality risk in patients with hematological malignancies is around 34%, although a study by Vijenthira et al. found the most relevant factor impacting mortality to be age [13]. COVID-19-positive leukemia patients have an increased fatality rate compared to patients with other cancer types [14], likely due to the susceptibility of blood cancer patients to experience lymphocyte depletion. Furthermore, anti-cancer treatments like cytotoxic chemotherapy can result in decreased T and B lymphocytes [15,16], leaving patients undergoing treatment vulnerable to severe infections. It has also been observed that patients receiving anti-cancer therapy tend to have low platelet and/or decreased neutrophil counts [15]. Although some studies have reported neutropenia as a risk factor in COVID-19-positive hematological malignancy patients [16], others found no significant connection [17]. However, cancer patients often have hyperactivated IL-6 [5,7], which may be another factor affecting COVID-19 severity, as multiple studies have shown that elevated IL-6 concentrations are associated with poor COVID-19 outcomes [18–20].

Immunosuppressed patients, such as solid organ transplant recipients, are treated with anti-T or anti-B cell therapies to prevent immunological rejection of transplantable tissue. This results in decreased lymphocytes [3]. A recent study showed that IL-6 concentrations in COVID-19 immunosuppressed patients without autoimmune disease were significantly increased compared to COVID-19 patients without immunosuppression [4]. Together, this lack of lymphocytes and elevated concentrations of inflammatory cytokines (i.e., IL-6) results in a weakened immune response against acute infections including ones caused by respiratory viruses, resulting in severe infections [21]. IL-6 dysregulation may result in hyperinflammation that is characteristic of severe COVID-19, particularly in patients requiring intensive care [22].

Once it was identified that extreme inflammatory responses could develop from SARS-CoV-2 infections, potential causes and treatment strategies were intensively studied [22–24]. Although the direct causes of hyperinflammation have yet to be established, several hypotheses exist [22]. One links the condition with the viral replication leading to pyroptosis, a highly inflammatory form of apoptosis, which then causes a pro-inflammatory cytokine reaction that affects macrophages and lymphocytes [22] and causes excessive IL-6 production [25]. Others include uncontrolled adaptive and neutralizing antibody responses, proposing that antibody binding to spike protein causes hyperinflammation [22]. Collecting longitudinal data in humans, particularly vulnerable populations, can be difficult and thus limited in scope. Further, these studies may not be able to uncover kinetic differences and causes for dysregulated immune responses, which are difficult to establish in humans, particularly given that early infection dynamics are generally not captured in clinical data. Mechanistic mathematical modelling helps to overcome these complexities because it allows for the investigation of immune response mechanisms, aids the prediction of clinical outcomes or vaccination efficacy [26,27], and facilitates uncovering potential drivers of severity with limited data sources [28].

To study the immune response and effects of antiviral drug therapies to SARS-CoV-2 infections, multiple studies deployed within-host mathematical modelling [29–39]. For example, Ghosh et al. [29] developed a within-host model that incorporates both innate and adaptive immune responses. The model was calibrated using viral load data from two COVID-19 patients and used to assess antiviral drug efficacy. Kim et al. [30] used a model of viral dynamics based on a target-cell limited model to investigate the effects of antiviral therapy on SARS-CoV-2. They fit their model to longitudinal viral load data, and their results suggested that early drug administration (2–3 days before the viral load peak) is crucial for the therapy to be effective. Zhang et al. [31] leveraged within-host mathematical modeling to investigate the effects of monoclonal antibody combinations that block viral entry by binding to the SARS-CoV-2 spike protein. Antiviral therapy was incorporated by adjusting the viral infectivity parameter, resulting in fewer infected targeted cells. The authors showed that early initiation of antiviral therapy resulted in significant reduction of viral load peak and shortened the viral shedding. Bai et al. [32] introduced within-host mathematical model that predicted a 56% reduction in viral replication caused by molnupiravir therapy. Al-Darabsah et al. [33], used a within-host mathematical modeling to compare the effects of monoclonal antibodies, antivirals, and combinations therapy. Their results indicated that monoclonal antibodies were more effective in the early stage of infection, whereas antiviral drugs provided better long-term effects in viral reduction. Using a within-host model, Phan et al. [34] examined the impact of timing and duration of combination nirmatrelvir/ritonavir therapy on post-treatment viral rebound. They found that early treatment may preserve target cells. Further, their model predicted that the likelihood of rebound is increased if viral clearance was incomplete, possibly due to a delayed adaptive immune response; extending treatment to 10 days could substantially reduce this risk. Lastly, Goyal et al. [35] used within host mathematical modeling to study different antiviral strategies, including remdesivir, selinexor, neutralizing antibodies, and cellular immunotherapy. Their results suggested that early treatment can result in reduced viral shedding duration with minimal effects on the total amount of virus present in body over time. The latter was predicted to be lowered only if therapy was introduced in the presymptomatic phase of infection.

Here, we focused on investigating and predicting COVID-19 immune dynamics in vulnerable populations, including those undergoing cancer treatments or who are immunosuppressed without autoimmune disease (e.g., solid organ transplant recipients on immunosuppressive agents). For this, we extended the approach used in Jenner et al. [28], where we generated a cohort of COVID-19-positive virtual patients based on a mechanistic model of the immune response to SARS-CoV-2. Model parameters were estimated through a literature review, or by fitting to available cytokine (IFN-β, GM-CSF and IL-6, IFN-γ), viral load, and immune cell data. Initial cytokine and immune cell concentrations were derived from human plasma and lung tissue. Cytokine binding/unbinding kinetics and immune cell dynamics were obtained from literature [40–47] and previous modelling studies [40,48,49]. Viral parameters, such as the production, viral decay and infectivity were estimated based on SARS-CoV-2 viral load data from macaques [50] and hospitalized patients [51,52]. Some of the parameters were obtained through fitting sigmoidal effect curves [53] to experimental data (in vivo and in vitro) - for example, the production of IL-6 by infected cells was estimated based on *in vitro* data from Ye et al. [54]; the rate at which macrophages clear extracellular virus was obtained from *in vitro* measurements of macrophage-induced phagocyosis of foot-and-mouth disease virus from Rigden et al. [55]; and the rate of GM-CSF production by monocytes was estimated from Lee et al. [56]. Other parameters were estimated through sub-models [28] with ensuring to match observed patients responses. The remaining subset of parameters were defined in a way to maintain homeostasis using values from prior studies or quantitative approximations [28] (if data were not available). Next, a local sensitivity analysis, performed by varying these parameter values within ±20% of their baseline values, was used to identify those most significantly impacting on key model outputs (maximum viral load, maximum number of dead cells, minimum uninfected tissue, maximum number of inflammatory macrophages, maximum number of CD8 + T cells, maximum unbound IL-6, maximum unbound IFN, total exposure to type I IFN and time of IFN peak).

The most significant parameters (monocyte-to-macrophage differentiation by IL-6 ($p_{M_{\phi_{I},L}}$), IL-6 production by inflammatory macrophages ($p_{{L,M}_{\phi }}$), IFN production rate by infected cells ($p_{F,I}$), monocyte recruitment by infected cells ($p_{M,I}$), cell-related IC50 concentration of IFN on virus production ($\in_{F,I}$), EC50 concentration of inflammatory macrophages on the IFN production ($\eta_{F,M_{\phi I}}$) and IFN production by monocytes ($p_{F,M}$)) were then used to create a virtual population of SARS-CoV-2 infected individuals. Each virtual patient, defined as a unique set of the most sensitive parameters, was predicted to have distinct immune response dynamics to SARS-CoV-2 infection. To explore determinants of severe responses, Jenner et al. [28] compared each patient’s immune response (e.g., maximum IL-6 or T cell concentration) to an ‘inflammation marker’ (Ψ) that quantified each virtual patient’s response with respect to the whole cohort. This inflammation marker, which measured a virtual patient’s maximum neutrophil and IL-6 concentrations, and the extent of lung tissue damage, successfully distinguished severe and mild responses. Using this approach, we showed in Jenner et al. [28] that virtual patients with severe outcomes are more likely to experience delayed IFN peaks and CD8 + T cell depletion.

Because our previous work did not take existing comorbidities into account, in this study we used the same model to generate three virtual patient cohorts: 1) a cohort of COVID-19 + patients with cancer, 2) a cohort of COVID-19 + immunosuppressed patients, and 3) a reference group of COVID-19 + patients without cancer or immunosuppression. The virtual patient cohorts were created based on data collected from Montréal, Canada area hospitals [57–59] and data available in the literature. Our simulations suggested that both severe cancer and immunosuppressed patients have decreased CD8 + T cells, elevated neutrophils, and IL-6 concentrations, and delayed IFN peaks. As in our previous work, we found these alterations to be driven by monocyte to macrophage differentiation and monocyte recruitment, consistent with experimental and clinical studies [60–62], suggesting these are host-intrinsic rather than driven by comorbidities. Overall, our findings suggest that suppressed CD8 + T cells, overproduction of IL-6, and delayed IFN peaks are correlated with disease severity in cancer and immunosuppressed patients with COVID-19, similar to previous results in COVID-19 severe virtual patients described in Jenner et al. [28]. However, we determined that the most severe outcomes in cancer and immunosuppressed virtual patients were characterized by more marked increases in elevated neutrophils during infection, higher rates of monocyte to macrophage differentiation by IL-6, and increased monocyte recruitment by infected cells. Thus, our study further highlights that immune dysfunction is heightened in immunocompromised patients, with potential consequences on COVID-19 severity, and identifies biomarkers driving this dysregulation.

## Methods

### Mathematical model of the immune response to SARS-CoV-2

**Fig 1:**
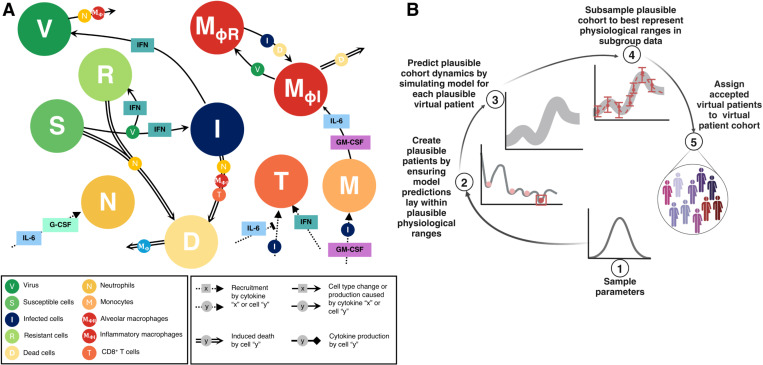
Mathematical model of the systemic immune response to SARS-CoV-2 and virtual patient generation algorithm. **A)** Mathematical model describing the immune response during COVID-19. Reproduced from Jenner et al. [28] under CC BY. The virus infects susceptible cells and creates infected or resistant cells based on IFN concentrations. Infected cells die and produce more virus or are eliminated by inflammatory macrophages, neutrophils (recruited by IL-6 and G-CSF), or CD8 + T cells whose population expands based on IFN concentrations and is inhibited by IL-6. Monocytes are recruited by infected cells and differentiate into inflammatory macrophages, which are regulated by GM-CSF and IL-6 concentrations. Some tissue-resident macrophages convert to become inflammatory after encountering infected or dead cells. See S1 Text for full model equations and parameter values. **B)** Schematic description of the VPC generation algorithm adapted from Jenner et al. [28]. 1) Parameters associated with macrophage, IL-6, and IFN production are sampled from normal distributions extracted from clinical data (Fig 2). 2) The model is simulated and simulated annealing is performed to minimize the distance between model predictions (outputs) and physiological ranges. 3) Virtual patients whose dynamics fit into the pre-defined ranges are assigned to the cohort of plausible patients. 4) The population of plausible virtual patients is subsampled based on data specific to each studied population (e.g., COVID-19 reference, cancer, immunosuppressed).

We used the differential equation-based mathematical model of Jenner et al. [28] that mimics the immune response to SARS-CoV-2 to understand and predict immune dynamics during COVID-19 (Fig 1A). The model describes the dynamics of immune cells (neutrophils, monocytes, CD8 + T cells, and tissue-resident and inflammatory macrophages) together with cytokine production and binding kinetics, including IFN-α,β, IL-6, granulocyte-macrophage colony-stimulating factor (GM-CSF), and granulocyte colony-stimulating factor (G-CSF).

In brief, the infection begins with the virus infecting susceptible lung epithelial cells (S), resulting in the production of virus (V) and infected cell (I) death. Infected cells can secrete IFN-α,β and, depending on the IFN concentration, neighbouring cells may also become resistant (R) to viral entry and replication. Infected cells are then removed by cytotoxic CD8 + T cells (T), inflammatory macrophages ($M_{\Phi I}$), and neutrophils (N), with neutrophils causing damage to all lung epithelial cells through their release of granules. Monocytes differentiate into inflammatory macrophages based on IL-6 and GM-CSF concentrations, and tissue-resident macrophages ($M_{\Phi R}$) can transition into inflammatory subsets through contact with either dead or infected cells. Dead cells (D) are eliminated by inflammatory macrophages. Neutrophils are recruited by IL-6 and G-CSF, whereas monocytes are attracted by infected cells and GM-CSF. Recruitment of CD8 + T cells is driven by infected cells and IFN and suppressed by IL-6 concentrations (it should be noted that we considered IL-6 as a proxy for the multitude of cytokines that inhibit T cell recruitment). A detailed description of the model equations and parametrization is provided in the S1 Text and Jenner et al. [28]. All model simulations were performed in Matlab [63] using *ddesd*.

### Generating virtual patient cohorts

**Fig 2:**
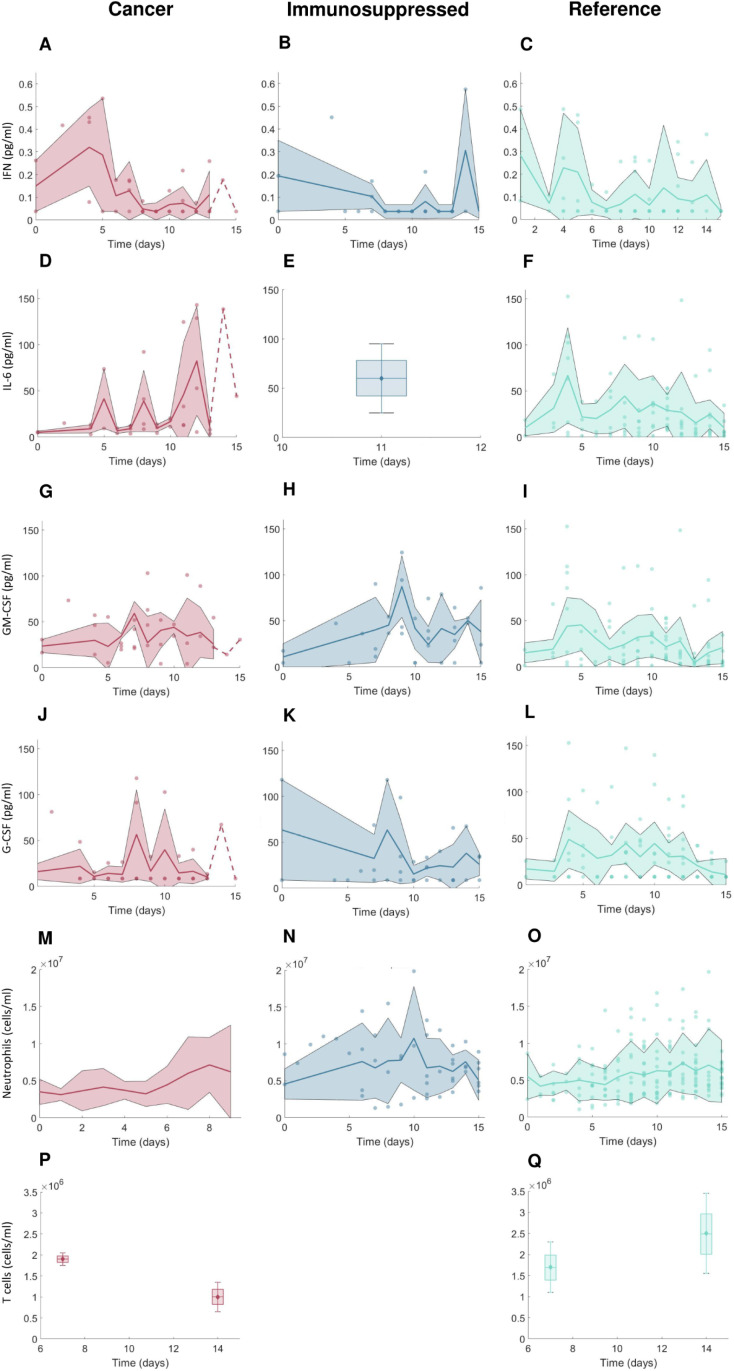
Clinical data measurements from 15 days post-symptom onset in COVID-19 + cancer patients, COVID-19 + immunosuppressed patients, and COVID-19 + patients without cancer or immunosuppression. A-C) IFN concentrations; D-F) IL-6 concentrations; G-I) GM-CSF concentrations; J-L) G-CSF concentrations; M-O) neutrophil concentrations; P-Q) CD8 + T cell concentrations. Note that there were no available CD8 + T cell data from immunosuppressed patients. For further descriptions of the clinical data used in our study, see the Methods and S1 Text. Solid lines: mean values. Shaded areas: standard deviations. Dashed lines indicate single observations. Boxplots were used where data were available only on certain days with mean values indicated by diamonds and standard deviations marked by error bars.


To generate the three virtual patient cohorts (VPCs) in our study, we followed the algorithm described in Jenner et al. [28] (Fig 1B) to ensure that each plausible patient’s immunological trajectory corresponded to available clinical data [4]. The generation process began by focusing on the most sensitive parameters (p) revealed in the sensitivity analysis including parameters associated with IFN, macrophage, and IL-6 production. All other model parameters were kept fixed. Varied parameters (p) were first sampled from normal distributions obtained from available clinical data [58], with mean values and standard deviations taken from measurements on specific days (see Fig 2). To define the normal distribution for each parameter, we selected a time point near the peak of the data, where the number of measurements was greatest. For each virtual patient, the model was simulated and the cost function [64],

$$\begin{array}{c} \min_{p}J\left(p\right)=\min_{p}\left[\sum_{i}\max\left({\left(M_{i}\left(p\right)-\frac{l_{i}+u_{i}}{2}\right)}^{2}-{\left(u_{i}-\frac{l_{i}+u_{i}}{2}\right)}^{2},0\right)\right], \end{array}$$ (1)

was minimized using simulated annealing. Here, $M_{i}\left(p\right)$ is the model output, and $l_{i}$ and $u_{i}$ are the lower and upper bounds of each immune population, respectively. We used simulated annealing via the *simulannealbnd* function in Matlab [65] for this optimization. If predicted dynamics fell within the established data ranges, a virtual patient was accepted as a plausible patient and placed into their respective virtual patient cohort (VPC). Data ranges were estimated based on the minimal and maximal values throughout the study period and were specific to each VPC.

Using this approach, we created three VPCs to represent hospitalized and outpatients with 1) COVID-19 and cancer (cancer VPC), 2) COVID-19 and immunosuppression (immunosuppressed VPC), and 3) COVID-19 without cancer or immunosuppression (reference VPC). We endeavoured to match the predicted dynamics in VPC to the corresponding patient data. To reduce the effects of any mismatch between predicted trajectories and our data, we verified that the trends observed in the immune responses of patients in our clinical trial (e.g., decreased T cell and increased IL-6 concentrations in more severe patients, neutropenia in cancer patients receiving cytotoxic chemotherapies) were also present in our VPCs. For this, we subsampled patients within each VPC to more tightly match available clinical [57–59] and reference data [4,5] (Fig 2; data descriptions can be found in the S1 Text). Because COVID-19-positive cancer and immunosuppressed patients tend to have fewer lymphocytes [3,5] and increased IL-6 [4,5] concentrations, we subsampled virtual patients according to the data from each of these patient groups. For the cancer VPC, we used data from Cai et al. [5] and clinical data from Montréal hospitals [57–59] for CD8 + T cells and IL-6 concentrations (Fig 2).”

To replicate the neutropenia experienced by patients undergoing chemotherapy [66], we decreased the initial concentrations of neutrophils for virtual patients in the cancer VPC. For this, we digitized the data from neutropenic patients during SARS-CoV-2 infection described in Lee et al. [66] using PlotDigitizer [67]. To generate immunosuppressed virtual patients, we used IL-6 concentrations from Monreal et al. [4]. Given their overall higher IL-6 concentrations, these virtual patients also experienced lower CD8 + T cell counts compared to those in the COVID-19 reference VPC, consistent with our model findings [28]. We assumed that these decreased values were representative of the CD8 + T cell dynamics in hospitalized patients, as CD8 + T cells are lower in immunosuppressed patients due to ongoing treatments. Though not all of our simulated trajectories match all data, we ensured that they corresponded to trends in our data and/or clinical measurements reported in the literature, when our data were insufficient or noisy (i.e., decreased T cell counts [68–70] and high IL-6 concentrations [71,72] in immunosuppressed patients). In all, this process resulted in the creation of 280 patients in each of the three VPCs.

### Evaluating disease severity across VPCs using an updated inflammation marker

To compare patient responses across VPCs, we modified the equation for the inflammation marker ($\Psi^{j}$) introduced by Jenner et al. [28] to evaluate the severity of COVID-19 + virtual patients. This inflammation marker measures each virtual patient’s maximum IL-6 and neutrophil concentrations, and maximal lung tissue damage (i.e., concentration of D, see S1 Text) according to the mean in the VPC. These patient attributes were chosen as they are known to be strongly associated with the final disease outcomes (i.e., disease severity). By comparing patient immune populations to the inflammation marker, our prior work found that IFN peaks were correlated with severity and that the IFN peak delay defined a severity threshold for $\Psi^{j}$ that separated mild from severe cases. Thus, it was concluded that patients with delayed IFN peaks (i.e., those with inflammation marker values above 3) experienced worse (more severe) outcomes.

In our previous work, patient severity was classified based on the inflammation marker ($\Psi^{j}$) values, but it was done within a single VPC. However, to compare patient responses *between* VPCs, adjustments to the inflammation marker equation were necessary, given that the normalization (denominator terms) in the original equation are specific to the VPC being considered and that these values will vary across VPCs. Thus, we opted here to use the COVID-19 reference VPC as a baseline to measure severity across VPCs. Accordingly, we modified the denominator values to reflect mean biomarker values from the reference VPC:


$$\Psi^{j}=\frac{\max_{t}\left(L_{U}^{j}(t)\right)}{\frac{1}{V^{ref}}\sum_{j^{ref}=1}^{V^{ref}}\left(\max_{t,j^{ref}}\left(L_{U}^{j^{ref}}(t)\right)\right)}+\frac{\max_{t}\left(N^{j}(t)\right)}{\frac{1}{V^{ref}}\sum_{j^{ref}=1}^{V^{ref}}\left(\max_{t,j^{ref}}\left(N^{j^{ref}}(t)\right)\right)}+\frac{S_{\max}-\min_{t}\left(S^{j}(t)+R^{j}(t)\right)}{\frac{1}{V^{ref}}\sum_{j^{ref}=1}^{V^{ref}}\left(S_{max}^{j}-\min_{t,j^{ref}}\left(S^{j^{ref}}(t)+R^{j^{ref}}(t)\right)\right)},$$ (2)

where the index j corresponds to the j-th patient, $L_{U}^{j}$, $N^{j}$, $S^{j}$ + $R^{j}$, and $\begin{array}{c} S_{max} \end{array}$ represent the concentrations of unbound IL-6, neutrophils, susceptible plus resistant epithelial cells (undamaged tissue), respectively, and $j^{ref}$ is the jth VP in the COVID-19 reference VPC with $V^{ref}$=280 being the total number of patients in the VPC. The change from our previous work allows for inter-VPC comparisons of patient responses, which is crucial as some patients in the vulnerable population VPC had lower inflammation marker ($\Psi^{j}$) values compared to COVID-19 reference patients while at the same time exhibiting markers of increased disease severity (e.g., higher IL-6, lower T cells, etc.) with respect to their VPC but not necessarily to the others. We then tested the updated inflammation marker against our data to validate its ability to distinguish between mild and severe responses based on clinical measurements. The marker effectively separated critical patients from those with less severe responses (S1 Fig).

### Sensitivity analysis

Given the neutropenic status of the cancer virtual patients in our study, in addition to the sensitivity analysis of full model parameters previously performed in Jenner et al. [28], we ran a local sensitivity analysis to see how changes in the initial concentration of neutrophils ($N_{0}$) may impact other populations and, thus, severity. For this, we varied the initial concentration of neutrophils from 60% to 140% of its baseline value and checked the differences in the output values of certain immune populations (maximal viral concentration, minimum tissue concentration, maximum IFN exposure, and maximum concentration of dead cells, inflammatory macrophages, CD8 + effector T cells, IL-6, IFN) compared to their baseline output values. Parameter variations that caused differences greater than 40% were considered significant.

### Comparing patients’ responses to the inflammation marker

To investigate the immune responses associated with disease severity, we compared a subset of each virtual patient’s immune characteristics (e.g., maximum T cell count, IFN peak) to their inflammation marker value. To identify potential relationships, we calculated Pearson’s correlation coefficient between these two values using the *corrcoef* function in Matlab [63]. We considered correlation coefficients of R ≥ 0.6 as indicative of moderate to strong correlations. Correlations were classified as statistically significant if p-values were less than 0.05.

### Statistical analysis

We used the Kolmogorov-Smirnov test at a level of significance of α=0.05 via the *kstest2* function in Matlab [63] to evaluate statistically significant pair-wise differences in the shape of distributions of virtual patient parameters between severe and mild patients across, and among VPCs. To analyze statistical differences in maximal biomarker values observed in VPCs, we performed ANOVA tests at a level of significance of α=0.05 using *anova1* function in Matlab [63]. To further analyze the statistical differences in parameter values and some maximal biomarker values (e.g., neutrophils, damaged tissue, and IFN concentrations) between certain groups of patients (i.e., severe versus mild), we performed a pairwise, non-parametric Wilcoxon test using the *stat_compare_means* function in R [73] and visualized the results using package *ggpubr*.

## Results

### Data suggest differences in immune biomarkers in cancer and immunosuppressed patients compared to those without comorbidities

From our clinical trial data, we found that cancer patients with COVID-19 tended to have decreased T cell count throughout the infection compared to cancer-free (reference) individuals with COVID-19 (Fig 2P-2Q). Compared to those without comorbidities, both cancer and immunosuppressed COVID-19 + patients had higher mean IL-6 concentrations after day 10 post symptom-onset, reaching above 60 pg/ml. The initial value of neutrophils was lowest in COVID-19 + cancer patients, but neutrophil concentrations were similar across the three groups throughout the infection. Mean values of GM-CSF were highest in the COVID-19 + immunosuppressed patients (Fig 2G-2I), whereas IFN values tended to decrease in cancer patients during infection, while they remained generally on a constant level in the reference VPC (Fig 2A-2C).

### Key differences in immunological dynamics of virtual patients in the reference, cancer, and immunosuppressed VPCs

To uncover the potential causes of the biomarker differences we found between cancer and immunosuppressed populations with respect to COVID-19 severity, we generated VPCs each consisting of 280 virtual individuals with COVID-19 who were otherwise healthy (“reference”), had cancer, or were immunosuppressed. To ensure all virtual patients trajectories matched the clinical data, we subsampled them based on the biomarker measurements from clinical [57–59] and reference data [4,5] sources (Fig 2; see Generating virtual patient cohorts in the Methods and the S1 Text).

**Fig 3:**
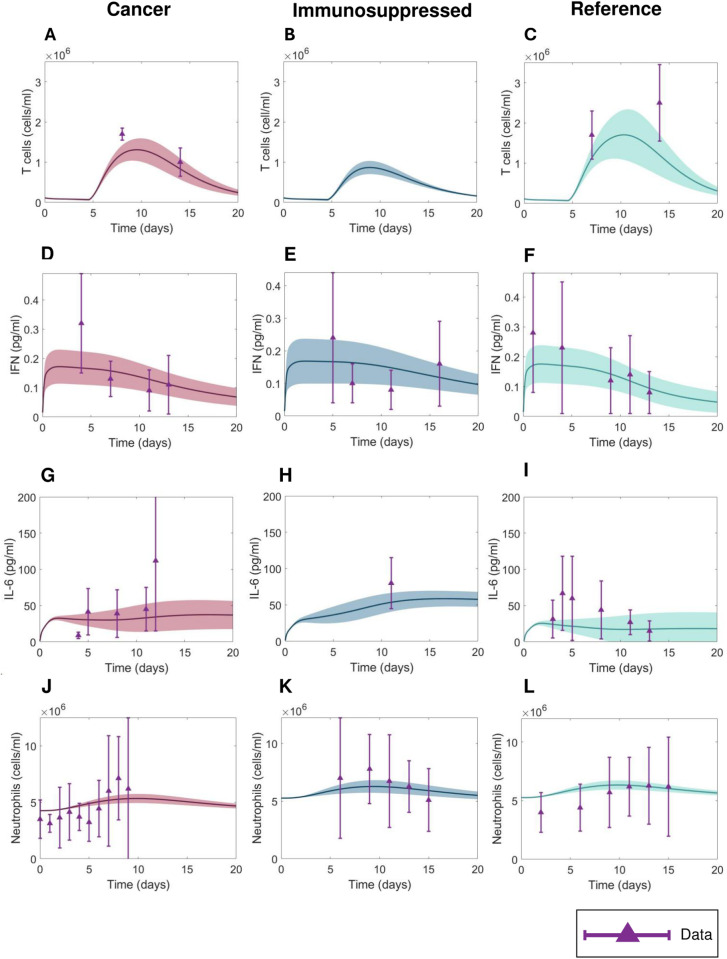
Comparison of immune dynamics in virtual patients from COVID-19 + cancer, immunosuppressed, reference VPCs with clinical data. A-C) CD8 + T cell dynamics. D-F) IFN dynamics. G-I) IL-6 dynamics. J-L) Neutrophils dynamics. Solid curves: mean values within each VPC. Shaded areas: standard deviations. Purple triangles: mean clinical values. Purple vertical lines: standard deviations from clinical observations (Fig 2 and S1 Text).

The virtual patient selection process (Fig 1B, see Methods) resulted in diverse dynamics. Namely, the cancer (Fig 3A) and immunosuppressed virtual patients (VPs) (Fig 3B) both exhibited significantly decreased CD8 + T cell concentrations (p < 10^-8^) compared to the VPs from the reference VPC (Fig 3C). Around 10 days after infection (when concentrations peaked), the mean T cell concentration reached $1.3\times 10^{6}$ cells/ml in the cancer VPC, while it was $1.8\times 10^{6}$ cells/ml in the COVID-19 reference VPC (Table 1). In comparison, VPs from the immunosuppressed VPC had lower maximal CD8 + T cell concentrations, with a mean of $0.9\times 10^{6}cells/ml$ (Table 1). These patterns also extended to IL-6 concentrations that similarly varied between VPCs: immunosuppressed patients had the highest maximal mean values of IL-6 of 60 pg/ml (Fig 3H, Table 1), followed by VPs in the cancer VPC (Fig 3G, Table 1), who were predicted to have an average peak value of 40 pg/ml. In contrast, virtual patients in the COVID-19 reference VPC had the lowest mean IL-6 peak concentrations of 25 pg/ml (Fig 3I, Table 1), which is consistent with reduced severity in otherwise healthy individuals. Statistical differences in maximal IL-6 values between three VPCs were confirmed by ANOVA (p < 10^-8^). A similar trend was observed in GM-CSF, where ANOVA confirmed statistically significant differences in maximal GM-CSF values between VPCs (p < 10^-8^); COVID-19 immunosuppressed virtual patients had highest mean GM-CSF maximal concentration (117 pg/ml), which was almost two times higher than in the COVID-19 reference VPC (60.17 pg/ml). By comparing maximal values of inflammatory macrophages, we also found statistical differences (p < 10^-8^) between all three VPCs. Maximal values of neutrophils were significantly decreased (p < 10^-8^) in the cancer VPC compared to other two VPCs (Fig 3J-3L, Table 1). Despite these dissimilarities, our model did not predict a statistically significant difference in the maximal IFN, G-CSF and monocyte concentrations between the three VPCs (Fig 3D-3F, Table 1).

**Table 1:** Predicted peak values over 20 days after infection onset for the three VPCs. Immunosuppressed VPs had the highest predicted maximal inflammatory macrophage, IL-6, and GM-CSF concentrations in addition to the lowest maximal CD8^+^ T cell concentrations. In comparison to the COVID-19 reference VPC, cancer virtual patients were predicted to have higher peak IL-6 and GM-CSF concentrations, increased inflammatory macrophages, and decreased maximal CD8^+^ T cells and neutrophils. Values indicate means and standard deviations (SD).

	Units	COVID-19 + cancer mean (SD)	COVID-19 + immunosuppressed mean (SD)	COVID-19 reference mean (SD)
**CD8** ^** +**^ ** T cells**	$10^{6}$cells/ml	1.32 (0.28)**	0.87 (0.17)**	1.78 (0.58)
**IFN**	pg/ml	0.17 (0.06)	0.17 (0.07)	0.18 (0.06)
**IL-6**	pg/ml	39.65 (12.95)**	58.75 (11.01)**	25.15 (12.25)
**Neutrophils**	$10^{6}$cells/ml	5.35 (0.39)**	6.29 (0.52)*	6.33 (0.36)
**GM-CSF**	pg/ml	78.92 (25.81)**	117.01 (21.89)**	60.17 (24.43)
**G-CSF**	pg/ml	27.27 (0.83)	27.18 (1.09)	27.25 (0.76)
**Monocytes**	$10^{5}$ cells/ml	4.59 (0.21)	4.59 (0.29)	4.58 (0.20)
**Inflammatory macrophages**	$10^{5}$cells/ml	1.83 (1.52)**	4.38 (2.72)**	1.51 (1.53)

*indicates a statistically significant difference (ANOVA) in maximal biomarker values found in patients from cancer and immunosuppressed VPCs compared to the reference VPC. Statistically significant differences found in maximal biomarker values in patients from cancer versus immunosuppressed VPCs are marked by * (p < 0.05).

### High tissue damage and increased occurrence of IFN peak delay characterize severe COVID-19 in immunosuppressed virtual patients

**Fig 4:**
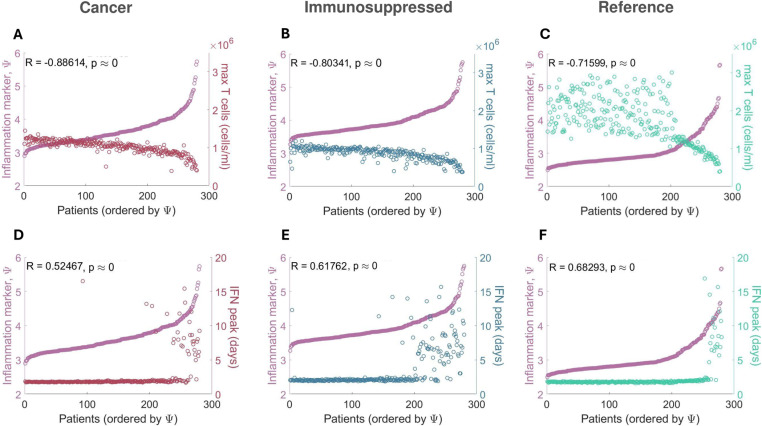
Correlations between maximal T cell and peak IFN concentrations and COVID-19 severity. Maximal T cell concentrations compared to the inflammation marker in patients from the **A)** COVID-19 + cancer VPC, **B)** COVID-19 + immunosuppressed VPC, and **C)** COVID-19 + reference VPC. Maximal CD8 + T cell concentrations in all three VPCs were found to be negatively correlated with the inflammation marker ($\Psi^{j}$). Time to IFN peak concentrations compared to the inflammation marker in patients from the **D)** COVID-19 + cancer VPC, **E)** COVID-19 + immunosuppressed VPC, and **F)** COVID-19 + reference VPC. IFN peak times were positively correlated with the inflammation marker ($\Psi^{j}$) in the immunosuppressed and COVID-19 reference VPCs. Patients were ordered by their inflammation maker ($\Psi^{j}$) values, with the mildest patients having the lowest inflammation marker ($\Psi^{j}$) values and the most severe the highest inflammation marker ($\Psi^{j}$) values.

To uncover mechanistic differences in immune responses in mild and severe COVID-19 virtual patients from vulnerable populations, we compared characteristics (e.g., maximal T cell concentrations) across our three VPCs using our updated inflammation marker (Eq. (2)). In all three VPCs, severe patients (patients with high values of severity marker [$\Psi^{j}$]) tended to have depleted CD8 + T cells (Fig 4A-4C) with the strongest negative correlation (R = -0.88, p < 10^-8^) with the inflammation marker ($\Psi^{j}$) found in the cancer VPC. A strong positive correlation (R > 0.9, p < 10^-8^) was observed between inflammation marker ($\Psi^{j}$) and maximal IL-6 concentrations (S2A-2C Fig), and the maximal concentration of inflammatory macrophages (R > 0.85, p < 10^-8^; S2D-2F Fig). In the cancer and immunosuppressed VPCs, we also found a statistically significant weak correlation between severity and peak neutrophil concentrations (R ≈ 0.4, p < 10^-8^) in addition to the degree of lung tissue damage (R ≈ 0.5, p-value < 10^-8^) while in the COVID-19 reference VPC, no such relationships were established (maximum neutrophils: R = 0.064, p-value = 0.288; maximum damaged lung tissue: R = -0.108, p = 0.072; see S2G-2I and S2J-2L Figs). Moderate correlations (R > 0.6, p < 10^-8^) between the inflammation marker ($\Psi^{j}$) and peak IFN concentrations were found in both the immunosuppressed and COVID-19 reference VPCs (Fig 4E-4F), with severe immunosuppressed patients ($\Psi^{j}$ > 4) having IFN peak delays more often than patients from other VPCs. In the cancer VPC, we only observed a statistically significant but weak correlation (R < 0.6; p < 10^-8^) between the IFN peak and the inflammation marker ($\Psi^{j}$). Together, these findings suggest increased immunological dysregulation in cancer and immunosuppressed virtual patients.

### Increased monocyte recruitment rates indicate more innate immune dysregulation in vulnerable patients versus reference patients

**Fig 5:**
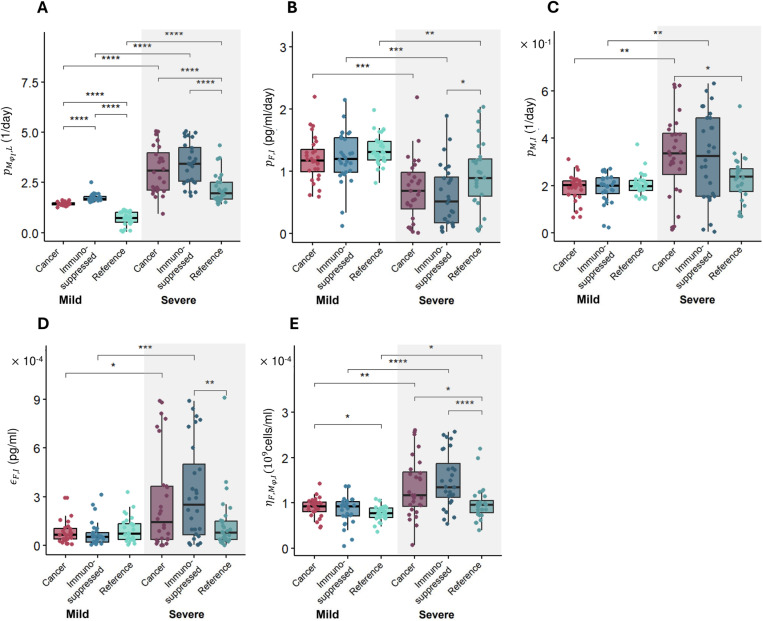
Differences in parameter values between mild and severe patients in each VPC. Differences in parameters associated with **A)** monocyte-to-macrophage differentiation by IL-6 ($p_{M_{\phi I},L}$), **B)** IFN production rate by infected cells ($p_{F,I}$), **C)** monocyte recruitment by infected cells ($p_{M,I}$), **D)** cell-related IC50 concentration of IFN on virus production ($\in_{F,I}$), and **E)** EC50 concentration of inflammatory macrophages on the IFN production ($\eta_{F,M_{\phi I}}$)*.* Box plots show the mean values of parameters in each VPC. Statistical significance in parameter values was assessed using a pairwise non-parametric Wilcoxon test (see Methods). *, p < 0.05; **, p < 0.005; ***, p < 0.0005; ****, p < 5e-5.

We next sought to identify the factors characterizing the least and most severe responses in all patient types and what metrics distinguish mild and severe disease in cancer and immunosuppressed patients. Testing these differences allows for the identification of factors that characterise most severe responses in all patients, and to establish whether there are additional factors that distinguish disease severity in cancer and immunosuppressed patients. Ultimately, these severity drivers could be used to improve the diagnosis, treatment, and stratification of at-risk groups. We selected virtual patients representing the lowest (mild) and highest (severe) 10% of the inflammation marker values in each VPC (more precisely, we chose 10% of patients in each VPC with lowest inflammation marker values and labeled them as ‘mild’, and the 10% of patients with the highest $\Psi^{j}$ values, who were labeled as ‘severe’) and performed statistical analyses using a pairwise non-parametric Wilcoxon test. The results suggest that there were increases (p < 10^-8^) in the mean values of parameters associated with monocyte-to-macrophage differentiation by IL-6 ($p_{M_{\phi_{I},L}}$; Fig 5A) and decreases (p < 0.05) in IFN production rates by infected cells ($p_{F,I}$; Fig 5B) in severe patients in all three VPCs. In particular, the values of $p_{M,I}$ (monocyte recruitment by infected cells; Fig 5C) and $\in_{F,I}$ (cell-related half-maximal inhibitory (IC_50_) concentration of IFN on the virus production; Fig 5D) were elevated only in the cancer and immunosuppressed virtual patients with severe COVID-19 (p < 0.05); differences between mild and severe virtual patients in the reference VPC were not observed. Values of $\eta_{F,M_{\phi I}}$ (half-maximal stimulatory (EC_50_) concentration of inflammatory macrophages on the IFN production; Fig 5E) were increased in cancer and immunosuppressed severe patients, but only in the latter VPC was the difference statistically significant (p < 0.05).

**Fig 6:**
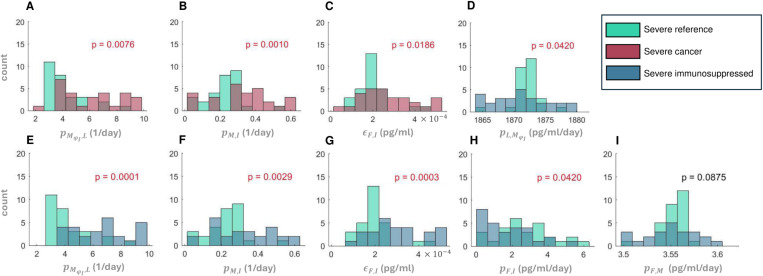
Statistical differences in parameter distributions between severe virtual patients. Statistically significant parameter distributions were evaluated by comparison to the COVID-19 reference VPC using a Kolmogorov-Smirnov test at a level of significance of α = 0.05. Comparison of the COVID-19 reference to **A-C)** severe cancer and **D-I)** severe immunosuppressed. The parameter being compared is denoted on the horizontal axis. **A)** and **E)** $p_{M_{\phi I},L}$ (monocyte-to-macrophage differentiation by IL-6). **B)** and **F)** $p_{M,I}$ (monocyte recruitment by infected cells). **C)** and **G)** $\in_{F,I}$ (cell-related IC50 concentration of IFN on the virus production). **D)** $p_{L,M_{\phi I}}$ (IL-6 production by inflammatory macrophages). **H)** $p_{F,I}$ (IFN production rates by infected cells). **I)** $\eta_{F,M_{\phi I}}$ (EC50 concentration of inflammatory macrophages on the IFN production). Red p*-*values indicate statistically significant differences between parameter distributions.

We then performed a Kolmogorov-Smirnov test to check for statistically significant differences in parameter distributions between the mildest 10% and most severe 10% of virtual patients in all three VPCs (S3-10 Figs). Three parameters from the cancer VPC were found to differ from the reference VPC when considering the severe virtual patients (Fig 6A-6C). These included the rate of monocyte-to-macrophage differentiation by IL-6 ($p_{M_{\phi_{I},L}}$), the rate of monocyte recruitment by infected cells ($p_{M,I}$), and the IC50 concentration of IFN on virus production ($\in_{F,I}$). We also found statistically different distributions of six parameters between the severe immunosuppressed and COVID-19 + reference VPs (Fig 6D-6I), again supporting the observation of increased immune dysregulation in these vulnerable populations. To further characterize the degree of these immunological differences, we also compared parameter distributions between the cancer and immunosuppressed VPCs and found a statistically significant difference in only one parameter ($p_{M_{\phi_{I},L}}$*,* the monocyte-to-macrophage differentiation by IL-6) between mild patients (S9 Fig) and no statistical differences between severe patients in those two VPCs (S10 Fig).

### Elevated neutrophils are associated with the highest tissue damage in severe cancer and immunosuppressed patients

**Fig 7:**
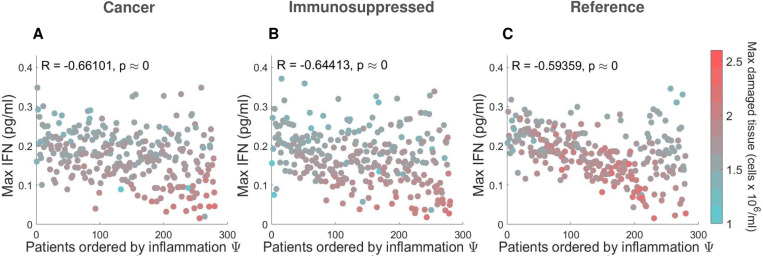
Relationships between maximal IFN and damaged tissue concentrations in virtual patients ordered by severity. **A)** COVID-19 + patients with cancer. **B)** COVID-19 +** **patients with immunosuppression. **C)** COVID-19 + reference patients. In all three VPCs, maximum IFN concentrations were negatively correlated with the degree of damaged tissue. In the cancer and immunosuppressed VPCs **(A and B)**, the most severe patients (i.e., those with the highest inflammation marker values) were found to have the most tissue damage, contrary to virtual patients in the COVID-19 reference VPC **(C)**. Patients are ordered from the lowest to highest inflammation marker values ($\Psi^{j}$).

Using the adjusted inflammation marker, we also examined the relationship between cytokines, cells, and lung tissue damage to distinguish potential severity drivers within each VPC. Overall, we found no correlation between model variables, except for a negative correlation between maximal IFN and damaged tissue (R ≤ -0.6, p < 10^-8^) in the three VPCs (Fig 7A-7C). The highest degree of lung tissue damage (marked by red dots) was predicted in the most severe cancer and immunosuppressed patients (compared to mild ones), but not in the most severe patients in the COVID-19 reference VPC (Fig 7C).

**Fig 8:**
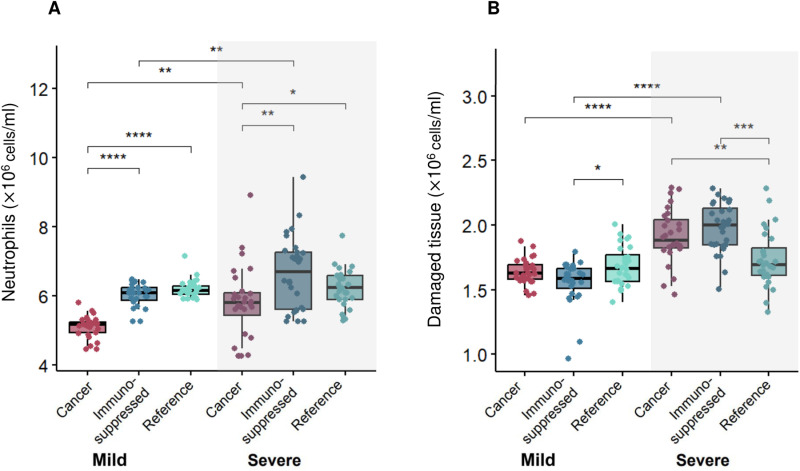
Relationships between maximum neutrophil concentrations and damaged tissue in mild and severe virtual patients. Mean values of **A)** maximum neutrophils and **B)** damaged tissue were found to be statistically significantly (p < 0.05) increased in cancer and immunosuppressed virtual patients with severe COVID-19 versus those with mild disease. Statistical tests were performed using a pairwise non-parametric Wilcoxon test (see Methods). *, p < 0.05; **, p < 0.005; ***, p < 0.0005; ****, p < 5e-5.

To uncover potential factors causing these differences, we analyzed the dynamics and the mean of maximum predicted values of the top 10% of patients (most severe) and bottom 10% (most mild) virtual patients. Our model predicted a comparable amount of damaged tissue over time in the case of severe patients (S13 Fig). In agreement with findings on lung tissue damage (Fig 7), mean values of maximum damaged tissue were found to be increased only in severe cancer and immunosuppressed patients (Fig 8B).

Next, we investigated the dynamics and mean maximum values of other immune populations to look for a potential cause of that feature. Mean values of maximum IFN were decreased in severe patients versus mild patients in all three VPCs (S14B Fig). Further, we found that neutrophils were highest in severe immunosuppressed and cancer patients, with this trend also observed in maximum tissue damage (increased values in severe cancer and immunosuppressed patients, Fig 8B) with respect to neutrophil concentrations (Fig 8A). To confirm this, we performed a pairwise non-parametric Wilcoxon test to check for statistical differences. Indeed, we found statistically significant differences in maximal damaged tissue and neutrophils values between mild and severe patients in the cancer and immunosuppressed VPCs (Fig 8), contrary to the reference VPC. While checking for differences in the maximum IFN concentrations, statistical tests confirmed the differences between mild and severe patients in all three VPCs (S14B Fig).

### Cancer virtual patients experience overall higher viral loads

**Fig 9:**
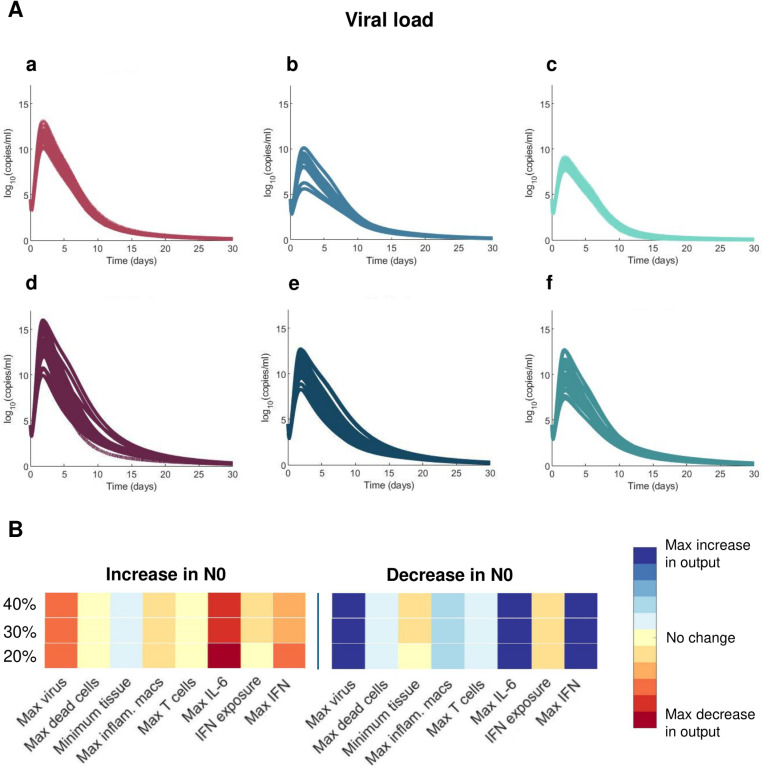
Cancer virtual patients with decreased initial neutrophil concentrations have higher viral load peaks. **A)** Viral loads in mild **a-c)** and severe virtual patients **d-f)**. **B)** Sensitivity analysis determined that decreasing *N*0 (the initial concentration of neutrophils) causes higher peak viral load and peak IL-6 and IFN concentrations.

Finally, our model predicted higher peak viral loads in both severe and mild cancer virtual patients as compared to virtual patients in the two other VPCs (Fig 9A). We hypothesized that this result was related to depressed initial neutrophil counts ($N_{0}$) in these virtual patients caused by chemotherapy-induced neutropenia [74]. To test this, we performed a sensitivity analysis by varying the initial concentration of neutrophils between 60% and 140% of its baseline value (see Methods). Decreasing the initial concentration of neutrophils ($N_{0}$) resulted in higher viral loads and maximum IL-6 and IFN concentrations (Fig 9B), seemingly confirming the assumed relationship between initial neutrophil concentrations and viral loads. This suggests that viral load is not the sole driver of severity, which is rather determined through a combination of immunological features [75].

## Discussion

A better understanding of the immune dynamics and potential causes of severe COVID-19 outcomes in vulnerable groups is essential to identifying the factors that drive the immune responses during COVID-19, reducing morbidity and mortality in these patients and selecting the best treatment courses. For example, patients with active cancers are more likely to experience worse COVID-19 disease outcomes as compared to those without cancer [76,77]. Similarly, individuals with immunosuppression triggered by lymphocyte-targeting therapies after organ transplantation have been reported to suffer from severe COVID-19 more often than immunosuppression-free patients [21]. Here, we used mathematical modelling and virtual patient cohorts to predict cellular immune response dynamics in COVID-19 in individuals with cancer or immunosuppression. Based on our previously developed mathematical model [28], we generated virtual patients whose immunological trajectories corresponded to clinical observations [4,5]. By comparing predicted outcomes between virtual patients with the lowest and highest inflammation marker values both between and within virtual patient cohorts, we distinguished biomarkers of immune dysregulation and severity, which has implications for drug development and clinical practices.

Our findings suggest that all severe COVID-19 patients, regardless of existing immunosuppression or cancer diagnoses, experience CD8 + T cell depletion, higher IL-6 concentrations, and importantly, delayed type I IFN peaks. Thus, these results further support the role of type I interferons in the control of SARS-CoV-2 infection severity [78]. We also observed delayed IFN peaks in some mild cancer and immunosuppressed virtual patients (S11 and S12 Figs). Relatedly, previous studies have found that IFN deficiency may be treated by anti-inflammatory therapies that target IL-6 [79]. Our model’s predictions further underline the major role of IL-6, which was found to be increased even in mild virtual patients in the cancer and immunosuppressed VPCs.

By comparing parameter values between the top and bottom 10% of virtual patients according to severity (i.e., severe versus mild), we found significant differences in five of the seven parameters used to generate the VPCs. Three of them (monocyte recruitment by infected cells, half-maximal stimulatory concentration of IFN production by inflammatory macrophages, and half-maximal inhibitory concentration of IFN on the virus production) were noticeably increased in severe cancer and immunosuppressed patients (Fig 5), suggesting their roles as potential severity indicators in those groups. Comparing the remaining two parameter values according to severity revealed differences between and within virtual patient cohorts. For example, the rate of monocyte-to-macrophage differentiation by IL-6 tended to be increased in severe patients and was highest in the immunosuppressed and cancer virtual patient cohorts and lowest in the COVID-19 reference virtual patient cohort. This agrees with clinical findings from circulating blood cells in the lungs in severe COVID-19 patients [80]. Further, IL-6 concentrations are higher in immunosuppressed patients [4], in agreement with our model predictions. The rate of IFN production by infected cells was also predicted to be highest in mild patients in the COVID-19 reference virtual patient cohort as compared to the other two virtual patient cohorts, again showing the significant role of IFN in coordinating a sufficient immune defense against SARS-CoV-2 infection.

Although neutrophils play a crucial role in blocking fungal and bacterial infections [81], their function in viral infection is not yet fully established [82]. When we decreased the initial neutrophil concentration to mimic the neutropenia characteristic of cancer patients, our model predicted higher peak viral loads in both severe and mild virtual cancer patients (Fig 9A). In our model, neutrophils quickly remove free viral particles and cause damage to all cells (including infected cells), hence a lower initial concentration of these cells may result in a higher number of infected cells leading to increased viral production at the beginning of infection. Later, when neutrophil concentrations in cancer virtual patients were predicted to reach comparable levels to virtual patients in the other virtual patient cohorts (around day 7 post-infection), we observed a similar trend in the viral load dynamics, namely that its concentration also decreased and was comparable to those predicted in the two other virtual patient cohorts. As a higher viral load peak was observed in severe patients, our results suggest that pre-existing neutropenia in cancer patients may be associated with adverse outcomes, consistent with findings from other studies [15]. However, throughout the infection, our model predicts that cancer patients with severe COVID-19 will nonetheless experience neutrophilia (Fig 8A), in agreement with clinical studies like that of Lee et al. [66].

Overall, our study supports the continued investigation of longitudinal immunological dynamics in groups vulnerable to COVID-19 by highlighting key mechanistic differences in their immune responses. In particular, our results revealed the effect of pre-existing neutropenia on viral load in cancer patients, which can result in a more severe course of infection [83]. However, we also found an association between elevated neutrophils and high tissue damage in severe cancer and immunosuppressed patients with COVID-19, which suggests the potential danger of neutrophilia even during immunosuppression. This may explain why some studies found no connection between neutropenia and severe COVID-19 [66], and even found decreased neutrophils beneficial. However, as other studies identified low neutrophil count as a potential risk factor [15] in COVID-19, our findings support considering neutropenia in treatment decisions. Interestingly, when considering full virtual patient cohorts, we did not find any correlations between maximum neutrophil count and damaged tissue. This lack of association is notable given the key roles of cell-mediated immunity during infection with SARS-CoV-2. For example, through the release of neutrophil extracellular traps and reactive oxygen species, neutrophils can cause extensive damage to tissues, so a correlation between maximum neutrophil count and damaged tissue would be expected in all patients. Moreover, by adjusting immunological trajectories to available CD8 + T cell and IL-6 data, our model predicted elevated pro-inflammatory compounds (such as GM-CSF) in both cancer and immunosuppressed patients. Thus, our results suggest the consideration of inhibitory therapies, as GM-CSF has been identified as a driver of lung tissue damage [84], and underline the delicate balance that must be struck to generate a robust yet controlled response to SARS-CoV-2. Together, this work puts forward a hypothesis for increased severity in both cancer and immunosuppressed patients, whose immunological systems are dysregulated either through disease or by immunomodulatory treatments.

Virtual populations based on mechanistic mathematical models enable the study and prediction of immune responses to viruses or vaccination without the need for extensive amounts of clinical data, making the approach a promising tool to study emerging infectious diseases and a variety of other contexts [28,85–88]. Nonetheless, our model has limitations. While our model captures several trends consistent with clinical observations, it does not always fully align with the available data. This may be a consequence of several factors. First, our dataset is sometimes sparse and lacks measurements of certain biomarkers in some patient subgroups (e.g., T cell data from immunosuppressed patients). Furthermore, it is possible that some biological mechanisms are not fully represented by the structure of our model. Other model structures may provide better fit to the data. However, as our model predictions display an overall consistency with our data and previous reports, we believe it nevertheless helps to uncover important dynamic patterns in immunological outcomes that support findings in the literature, even in the absence of rich data. Another limitation is that certain innate immune cells (i.e., natural killer cells [89]) and cytokines that play an important role in fighting SARS-CoV-2 (i.e., IL-1, IL-12, TNF-α [90]) were not considered in our model. However, the major model component IL-6, the main driver of T cell depletion throughout the infection, mimics the effects of other cytokines and drugs that inhibit T cell recruitment, suggesting our results can be extended to other anti-inflammatory cytokines not included in our model. Further, our model did not account for the humoral response provided by B cells and antibodies, and thus, it has a limited application to vaccination studies. Adding these components would enable the identification of other severity-associated factors and significantly improve our understanding of the intricate dynamics of the immune response to SARS-CoV-2 and other viral infections. However, immunocompromised patients often develop insufficient humoral immune responses to COVID-19 vaccination [11]. Even when achieved, these responses are typically short-lived. Additionally, in patients undergoing therapies with immunosuppressants after organ or stem cell transplantation, vaccination may not be effective because of an impaired B cell response [91,92], leaving these patients unable to produce antibodies. In particular, patients with lymphoid malignancies may not form any humoral immune defense after vaccination [93]. Lastly, our cohort included primary infections during the first waves of the COVID-19 pandemic. Hence, the patients did not have pre-existing immunity. Thus, in the context of this work, we expect excluding humoral responses to have little impact on our results. Nevertheless, incorporating B and antibody immunity into our model would enable the identification of the mechanisms of decreased vaccine efficacy, as reported in many studies [11,94], in vulnerable groups. Our study is also limited by the period of collection of the data used to generate our immunosuppressed and cancer VPCs. The clinical study used here provided data from primary infected patients during the first waves of the COVID-19 pandemic (March 2020-August 2020). However, though variants have evolved to be more transmissible between individuals, there are only modest or undetectable differences in viral loads after infections by these [95]. Additionally, CD8 + T cell-mediated protection after infection onset appears to follow similar dynamics across SARS-CoV-2 variants [96].

In summary, our findings corroborate that unregulated immune responses in cancer and immunosuppressed patients place them in a high-risk position of experiencing severe COVID-19. Furthermore, the approach presented here can be used in complement to experimental and clinical studies of COVID-19 and other viral respiratory diseases to comprehensively explore immune response kinetics after infection, thereby improving our understanding of the disease severity.

## Supporting information

S1 FigInflammation marker ($\Psi^{j}$) accurately separates critical COVID-19 + patients from those with moderate or severe disease.The values of $\Psi^{j}$ were computed using maximum neutrophil, IL-6, and RAGE concentrations measured at the day of peak in our hospitalized cohort. Statistically significant differences in the value of $\Psi^{j}$were found between patients with moderate and severe or moderate and critical disease. Significance was determined using a Wilcoxon test (*, p < 0.05; **, p < 0.005; ***, p < 0.0005; ****, p < 5e-5).(TIF)

S2 FigComparisons of the maximum IL-6, inflammatory macrophage, and neutrophil concentrations, and maximal tissue damage with the inflammation marker.Maximal IL-6 concentrations compared to the inflammation marker in patients from the A) COVID-19 + cancer VPC, B) COVID-19 + immunosuppressed VPC, and C) COVID-19 + reference VPC. Maximal IL-6 concentrations in all three VPCs were found to be positively correlated with the inflammation marker ($\Psi^{j}$). Maximal inflammatory macrophage values compared to the inflammation marker in patients from the D) COVID-19 + cancer VPC, E) COVID-19 + immunosuppressed VPC, and F) COVID-19 + reference VPC. Maximal inflammatory macrophages in all three VPCs were found to be positively correlated with $\Psi^{j}$. Maximal neutrophils compared to the inflammation marker in patients from the G) COVID-19 + cancer VPC, H) COVID-19 + immunosuppressed VPC, and I) COVID-19 + reference VPC. There was no correlation between maximal neutrophils and the inflammation marker in either of the three VPCs. Maximal damaged tissue compared to the inflammation marker in patients from the J) COVID-19 + cancer VPC, K) COVID-19 + immunosuppressed VPC, and L) COVID-19 + reference VPC. There was no correlation between maximal damaged tissue and the inflammation marker in any of the three VPCs (p $<10^{-10}$, indicating statistical significance).(TIF)

S3 FigParameter distribution comparisons between mild virtual patients in COVID-19 + cancer and COVID-19 + reference VPCs.A) Monocyte-to-macrophage differentiation by IL-6, B) IL-6 production by inflammatory macrophages, C) IFN production rates by infected cells, D) Monocyte recruitment by infected cells, E) EC50 concentration of inflammatory macrophages on the IFN production, F) Cell-related IC50 concentration of IFN on virus production, and G) IFN production by monocytes. Statistically significant differences were found for $p_{M_{\phi_{I}},L}$ and $\in_{F,I}$. Red p-values indicate statistically significant differences in distributions (p < 0.05).(TIF)

S4 FigParameter distribution comparisons between severe virtual patients in COVID-19 + cancer and COVID-19 + reference VPCs.A) Monocyte-to-macrophage differentiation by IL-6, B) IL-6 production by inflammatory macrophages, C) IFN production rates by infected cells, D) Monocyte recruitment by infected cells, E) EC50 concentration of inflammatory macrophages on the IFN production, F) Cell-related IC50 concentration of IFN on virus production, and G) IFN production by monocytes. Statistically significant differences were found for $p_{M_{\phi_{I}},L}$, $p_{F,I}$, $p_{M,I}$, $\eta_{F,M_{\phi I}}$, and $\in_{F,I}$. Red p-values indicate statistically significant differences in distributions (p < 0.05).(TIF)

S5 FigParameter distribution comparisons between mild virtual patients in COVID-19 + immunosuppressed and COVID-19 + reference VPCs.A) Monocyte-to-macrophage differentiation by IL-6, B) IL-6 production by inflammatory macrophages, C) IFN production rates by infected cells, D) Monocyte recruitment by infected cells, E) EC50 concentration of inflammatory macrophages on the IFN production, F) Cell-related IC50 concentration of IFN on virus production, and G) IFN production by monocytes. Statistically significant differences were found for $p_{M_{\phi_{I}},L}$ and $\in_{F,I}$. Red p-values indicate statistically significant differences in distributions (p < 0.05).(TIF)

S6 FigParameter distribution comparisons between severe virtual patients in COVID-19 + immunosuppressed and COVID-19 + reference VPCs.A) Monocyte-to-macrophage differentiation by IL-6, B) IL-6 production by inflammatory macrophages, C) IFN production rates by infected cells, D) Monocyte recruitment by infected cells, E) EC50 concentration of inflammatory macrophages on the IFN production, F) Cell-related IC50 concentration of IFN on virus production, and G) IFN production by monocytes. Statistically significant differences were found for $p_{M_{\phi_{I}},L}$, $p_{{L,M}_{\phi_{I}}}$, $p_{F,I}$, $p_{M,I}$, $\eta_{F,M_{\phi I}}$, and $\in_{F,I}$. Red p-values indicate statistically significant differences in distributions (p < 0.05).(TIF)

S7 FigParameter distribution comparisons between mild and severe virtual patients in the COVID-19 + cancer VPCs.A) Monocyte-to-macrophage differentiation by IL-6, B) IL-6 production by inflammatory macrophages, C) IFN production rates by infected cells, D) Monocyte recruitment by infected cells, E) EC50 concentration of inflammatory macrophages on the IFN production, F) Cell-related IC50 concentration of IFN on virus production, and G) IFN production by monocytes. Statistically significant differences were found for $p_{M_{\phi_{I}},L}$, $p_{F,I}$, $p_{M,I}$, $\eta_{F,M_{\phi I}}$, $\in_{F,I}$, and $p_{F,M}$. Red p-values indicate statistically significant differences in distributions (p < 0.05).(TIF)

S8 FigParameter distribution comparisons between mild and severe virtual patients in the COVID-19 + immunosuppressed VPCs.A) Monocyte-to-macrophage differentiation by IL-6, B) IL-6 production by inflammatory macrophages, C) IFN production rates by infected cells, D) Monocyte recruitment by infected cells, E) EC50 concentration of inflammatory macrophages on the IFN production, F) Cell-related IC50 concentration of IFN on virus production, and G) IFN production by monocytes. Statistically significant differences were found for $p_{M_{\phi_{I}},L}$, $p_{F,I}$, $p_{M,I}$, $\eta_{F,M_{\phi I}}$, and$\in_{F,I}$. Red p-values indicate statistically significant differences in distributions (p < 0.05).(TIF)

S9 FigParameter distribution comparisons between mild virtual patients in COVID-19 + cancer and COVID-19 + immunosuppressed VPCs.Rate of A) monocyte-to-macrophage differentiation by IL-6, B) IL-6 production by inflammatory macrophages, C) IFN production by infected cells, D) monocyte recruitment by infected cells, E) EC_50_ concentration of inflammatory macrophages on the IFN production, F) cell-related IC_50_ concentration of IFN on virus production, and G) IFN production by monocytes. Statistically significant differences were found for $p_{M_{\phi_{I}},L}$. Red p-values indicate statistically significant differences in distributions (p < 0.05).(TIF)

S10 Fig Statistical differences in parameter distributions between severe virtual patients in COVID-19 + cancer and COVID-19 + immunosuppressed VPCs. Rate of A) monocyte-to-macrophage differentiation by IL-6, B) IL-6 production by inflammatory macrophages, C) IFN production by infected cells, D) monocyte recruitment by infected cells, E) EC_50_ concentration of inflammatory macrophages on the IFN production, F) cell-related IC_50_ concentration of IFN on virus production, and G) IFN production by monocytes. Statistically significant differences were not found in any of the parameters. Red p-values indicate statistically significant differences in distributions (p < 0.05).
(TIF)

S11 Fig Relationships between maximum T cell and IL-6 concentrations and IFN peak. (A) Correlation between maximum T cell concentrations and maximum IL-6 in a) COVID-19 + cancer VPC, b) COVID-19 + immunosuppressed VPC, c) COVID-19 + reference VPC. (B) Model simulation for ‘outlier virtual patients’ (VP1, VP2, and VP3) showing a) viral loads, b) neutrophil concentrations, c) CD8 + T cells, and d) IL-6 concentrations.
(TIF)

S12 Fig Parameter value changes in outlier virtual patients provide insight into mechanisms of immune dysregulation in cancer virtual patients. A) Relationships between maximum IL-6, maximum T cell, and peak IFN concentrations in VP1, VP2, and VP3 in the COVID-19 + cancer VPC. B) Unbound IFN dynamics before and after decreasing values of parameters associated with IFN production. C) Uninfected cell dynamics of VP2 and VP3 before and after decreasing values of parameters associated with IFN production.
(TIF)

S13 Fig High tissue damage in severe patients in all three VPCs. Dynamics of damaged tissue in A) mild COVID-19 + cancer patients, B) mild COVID-19 + immunosuppressed patients, C) mild COVID-19 + reference patients, D) severe COVID-19 + cancer patients, E) severe COVID-19 + immunosuppressed patients, F) severe COVID-19 + reference patients.
(TIF)

S14 Fig Maximum IFN concentrations are decreased in severe patients. Dynamics of IFN in A: a) Mild COVID-19 + cancer patients, b) Mild COVID-19 + immunosuppressed patients, c) mild COVID-19 + reference patients, d) Severe COVID-19 + cancer patients, e) Severe COVID-19 + immunosuppressed patients, f) Severe COVID-19 + reference patients. B) Model predictions of mean values of maximal IFN concentrations in mild and severe virtual patients. Statistical differences in maximal IFN values between groups are marked by a level of significance (*) estimated based on p-values above the box plots (*, p < 0.05; **, p < 0.005; ***, p < 0.0005; ****, p < 5e-5). A pairwise non-parametric Wilcoxon test was used to assess statistical significance (see Methods).
(TIF)

S1 Text Table A. Viral kinetic parameters. **Table B.** Cell production, recruitment, and activation rates. **Table C.** Cell-related half-effect (EC50), IC50, and Hill coefficient (h) parameters. **Table D.** Cell- and virus-induced death rates. **Table E.** Cell death and virus decay rates. **Table F.** Cytokine production rates. **Table G.** Cytokine production regulation parameters. **Table H.** Cytokine linear clearance and internalization rates. **Table I.** Cytokine binding/unbinding rates and stoichiometric constants. **Table J.** Number of cellular receptors and cytokine molecular weights. **Table K.** Initial conditions. **Table L.** List of variables in the model equations. **Table M.** Parameter values of outlier virtual patients. Key immune response parameters were adjusted to analyze their impact on severity.
(DOCX)
