# Supplementary figures and images for: Using virtual patient cohorts to uncover immune response differences in cancer and immunosuppressed COVID-19 patients
Source: PLoS Comput Biol. 2025 Jun 9;21(6):e1013170. doi: 10.1371/journal.pcbi.1013170 (PMC12180667; doi:10.1371/journal.pcbi.1013170)

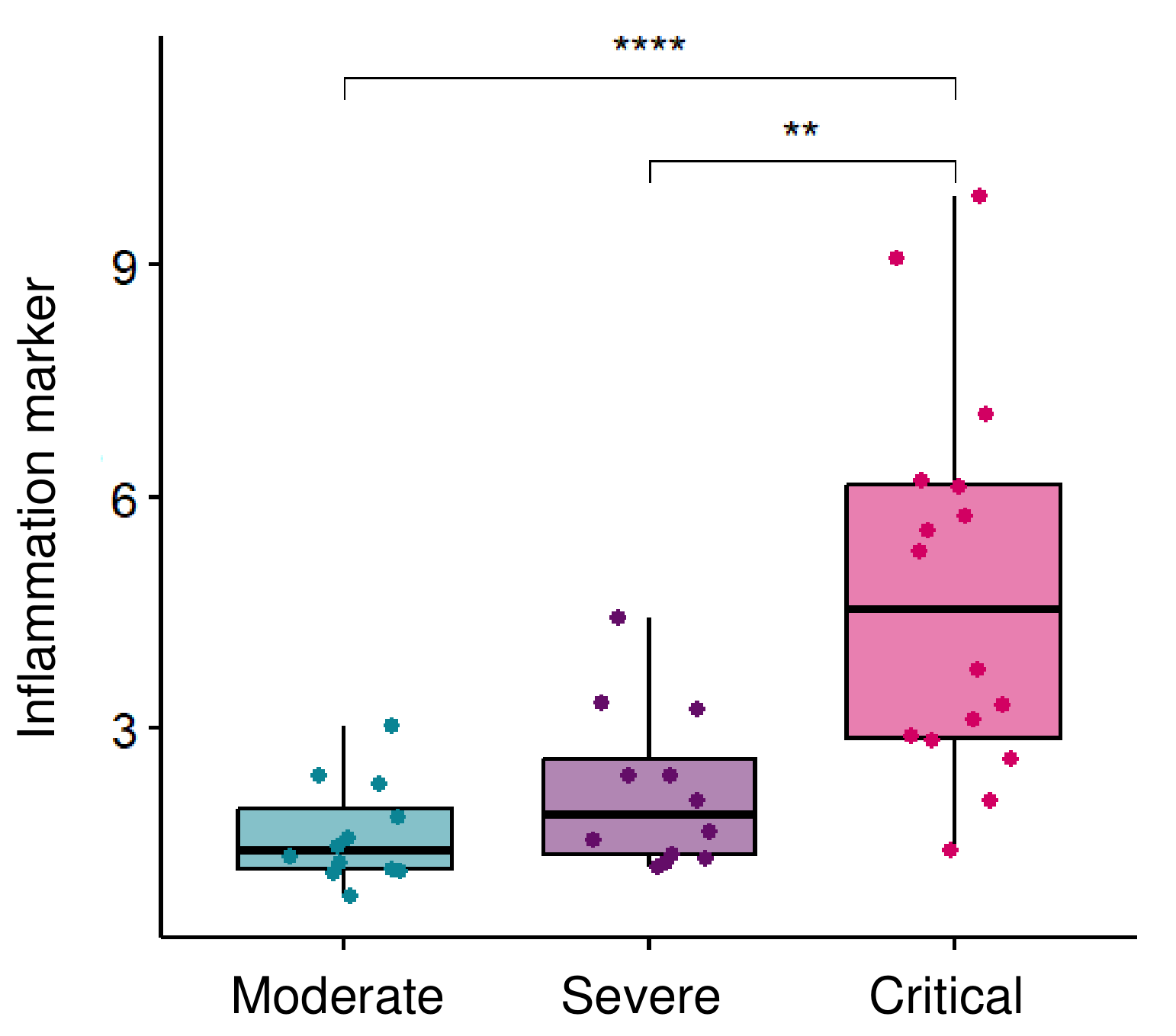

Supplement: S1 Fig — The values of Ψj were computed using maximum neutrophil, IL-6, and RAGE concentrations measured at the day of peak in our hospitalized cohort. Statistically significant differences in the value of Ψjwere found between patients with moderate and severe or moderate and critical disease. Significance was determined using a Wilcoxon test (*, p < 0.05; **, p < 0.005; ***, p < 0.0005; ****, p < 5e-5). (TIF) [file pcbi.1013170.s001.tif]

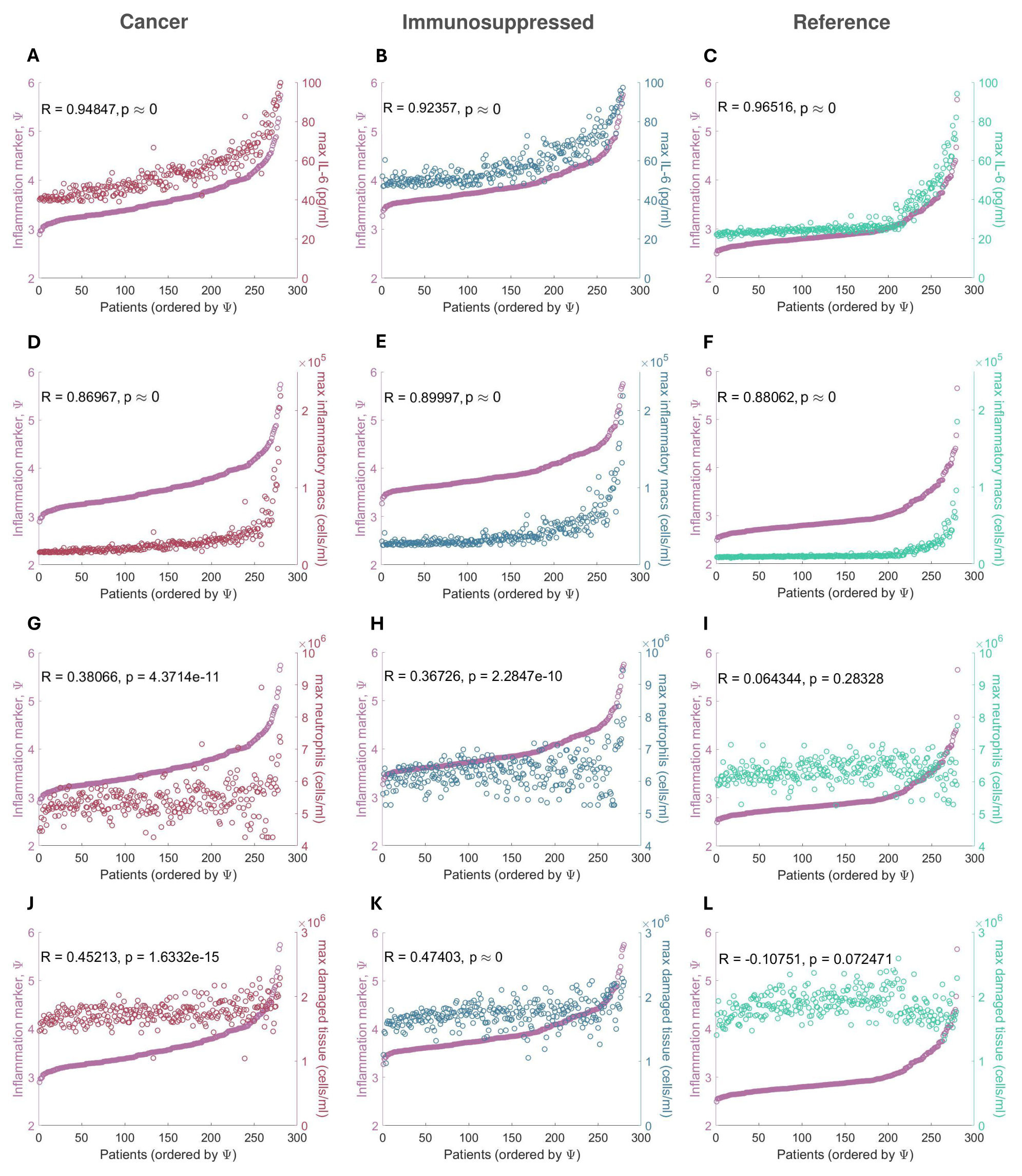

Supplement: S2 Fig — Maximal IL-6 concentrations compared to the inflammation marker in patients from the A) COVID-19 + cancer VPC, B) COVID-19 + immunosuppressed VPC, and C) COVID-19 + reference VPC. Maximal IL-6 concentrations in all three VPCs were found to be positively correlated with the inflammation marker (Ψj). Maximal inflammatory macrophage values compared to the inflammation marker in patients from the D) COVID-19 + cancer VPC, E) COVID-19 + immunosuppressed VPC, and F) COVID-19 + reference VPC. Maximal inflammatory macrophages in all three VPCs were found to be positively correlated with Ψj. Maximal neutrophils compared to the inflammation marker in patients from the G) COVID-19 + cancer VPC, H) COVID-19 + immunosuppressed VPC, and I) COVID-19 + reference VPC. There was no correlation between maximal neutrophils and the inflammation marker in either of the three VPCs. Maximal damaged tissue compared to the inflammation marker in patients from the J) COVID-19 + cancer VPC, K) COVID-19 + immunosuppressed VPC, and L) COVID-19 + reference VPC. There was no correlation between maximal damaged tissue and the inflammation marker in any of the three VPCs (p <10−10, indicating statistical significance). (TIF) [file pcbi.1013170.s002.tif]

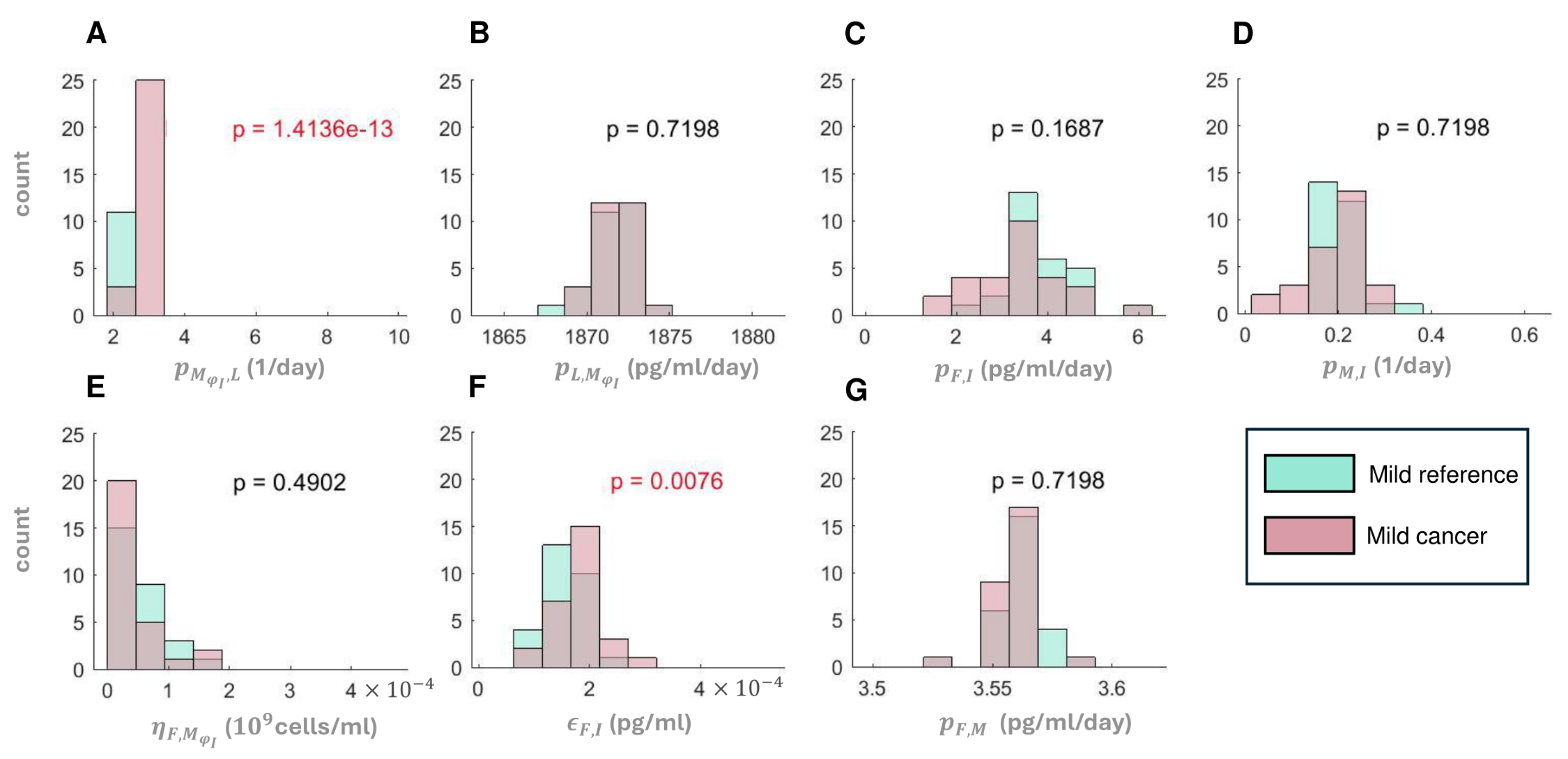

Supplement: S3 Fig — A) Monocyte-to-macrophage differentiation by IL-6, B) IL-6 production by inflammatory macrophages, C) IFN production rates by infected cells, D) Monocyte recruitment by infected cells, E) EC50 concentration of inflammatory macrophages on the IFN production, F) Cell-related IC50 concentration of IFN on virus production, and G) IFN production by monocytes. Statistically significant differences were found for pMϕI,L and ∈F,I. Red p-values indicate statistically significant differences in distributions (p < 0.05). (TIF) [file pcbi.1013170.s003.tif]

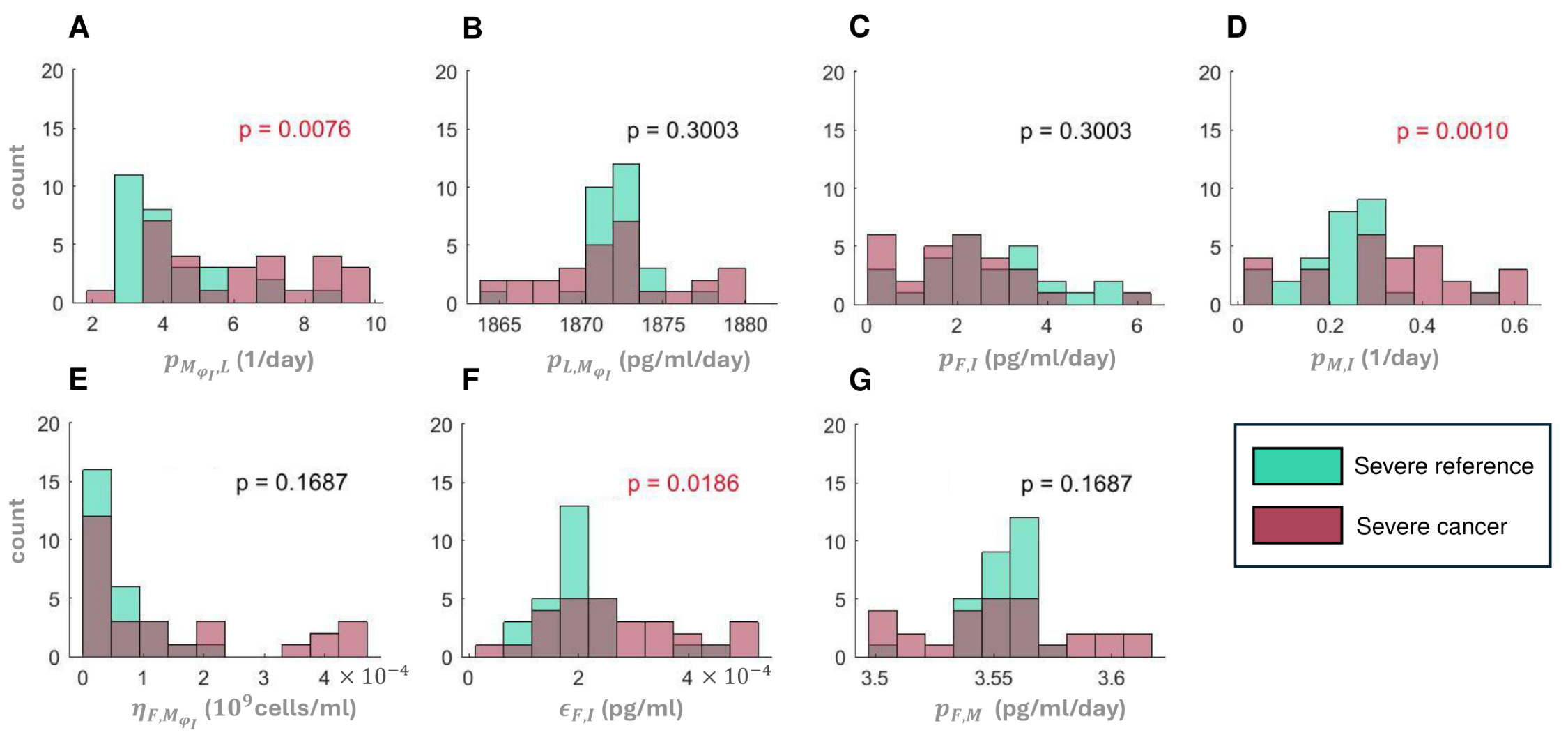

Supplement: S4 Fig — A) Monocyte-to-macrophage differentiation by IL-6, B) IL-6 production by inflammatory macrophages, C) IFN production rates by infected cells, D) Monocyte recruitment by infected cells, E) EC50 concentration of inflammatory macrophages on the IFN production, F) Cell-related IC50 concentration of IFN on virus production, and G) IFN production by monocytes. Statistically significant differences were found for pMϕI,L, pF,I, pM,I, ηF,MϕI, and ∈F,I. Red p-values indicate statistically significant differences in distributions (p < 0.05). (TIF) [file pcbi.1013170.s004.tif]

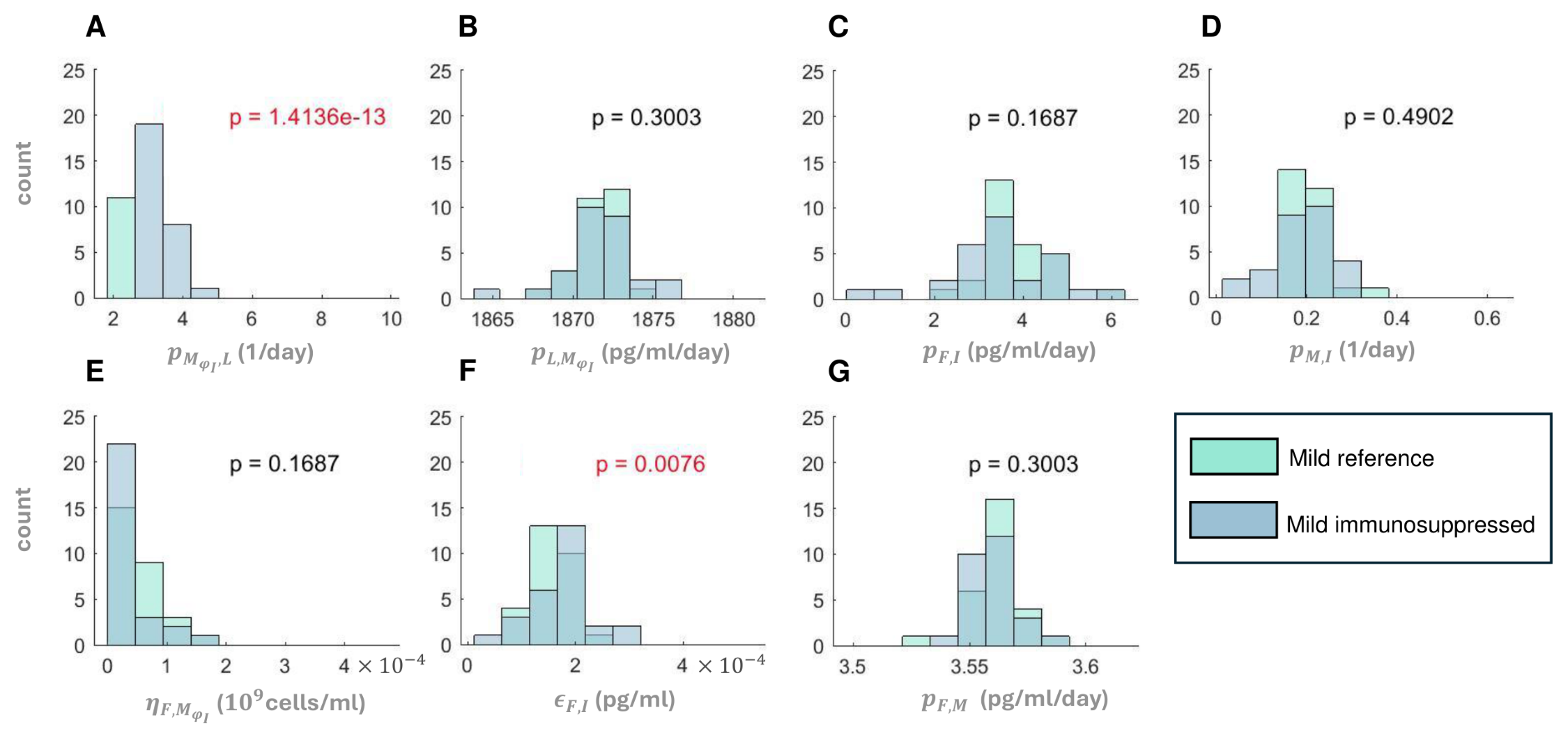

Supplement: S5 Fig — A) Monocyte-to-macrophage differentiation by IL-6, B) IL-6 production by inflammatory macrophages, C) IFN production rates by infected cells, D) Monocyte recruitment by infected cells, E) EC50 concentration of inflammatory macrophages on the IFN production, F) Cell-related IC50 concentration of IFN on virus production, and G) IFN production by monocytes. Statistically significant differences were found for pMϕI,L and ∈F,I. Red p-values indicate statistically significant differences in distributions (p < 0.05). (TIF) [file pcbi.1013170.s005.tif]

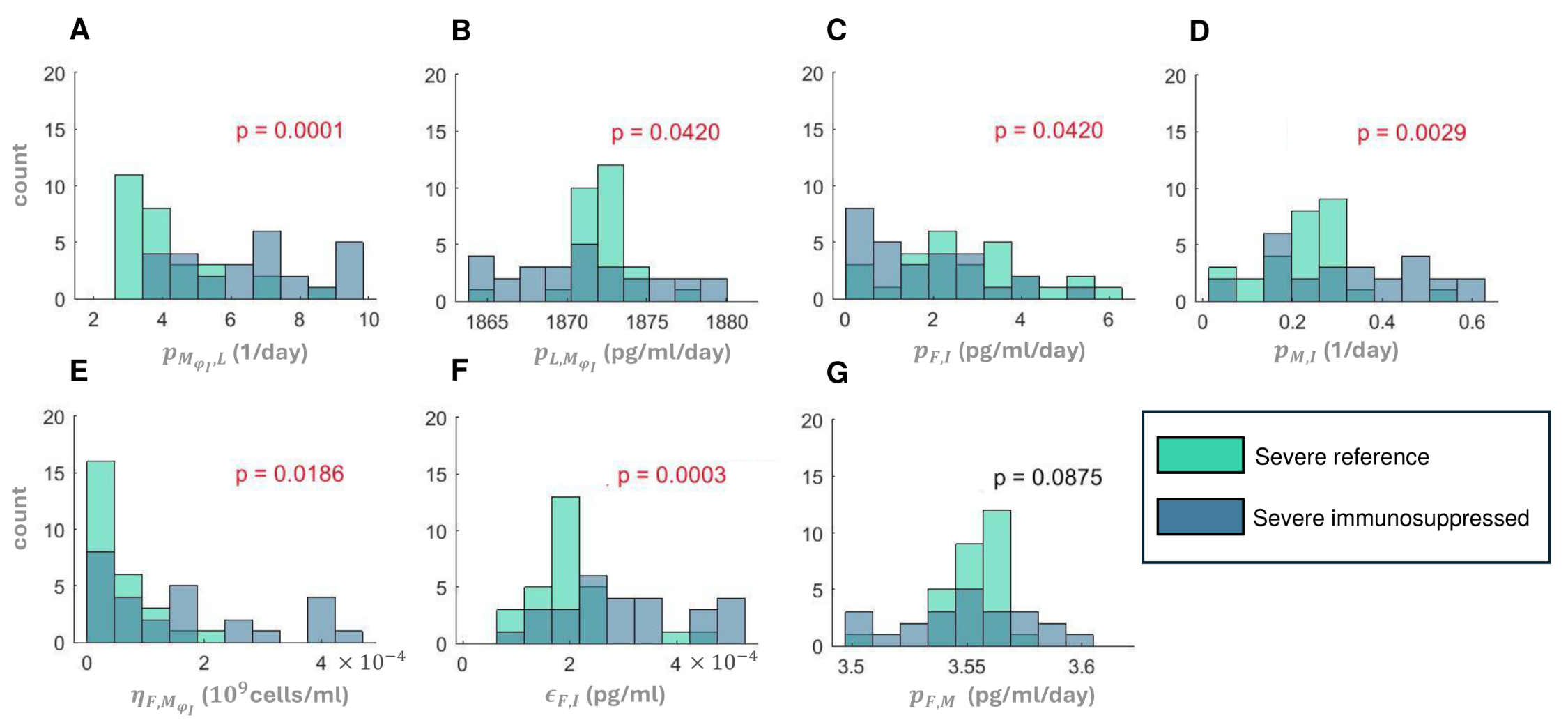

Supplement: S6 Fig — A) Monocyte-to-macrophage differentiation by IL-6, B) IL-6 production by inflammatory macrophages, C) IFN production rates by infected cells, D) Monocyte recruitment by infected cells, E) EC50 concentration of inflammatory macrophages on the IFN production, F) Cell-related IC50 concentration of IFN on virus production, and G) IFN production by monocytes. Statistically significant differences were found for pMϕI,L, pL,MϕI, pF,I, pM,I, ηF,MϕI, and ∈F,I. Red p-values indicate statistically significant differences in distributions (p < 0.05). (TIF) [file pcbi.1013170.s006.tif]

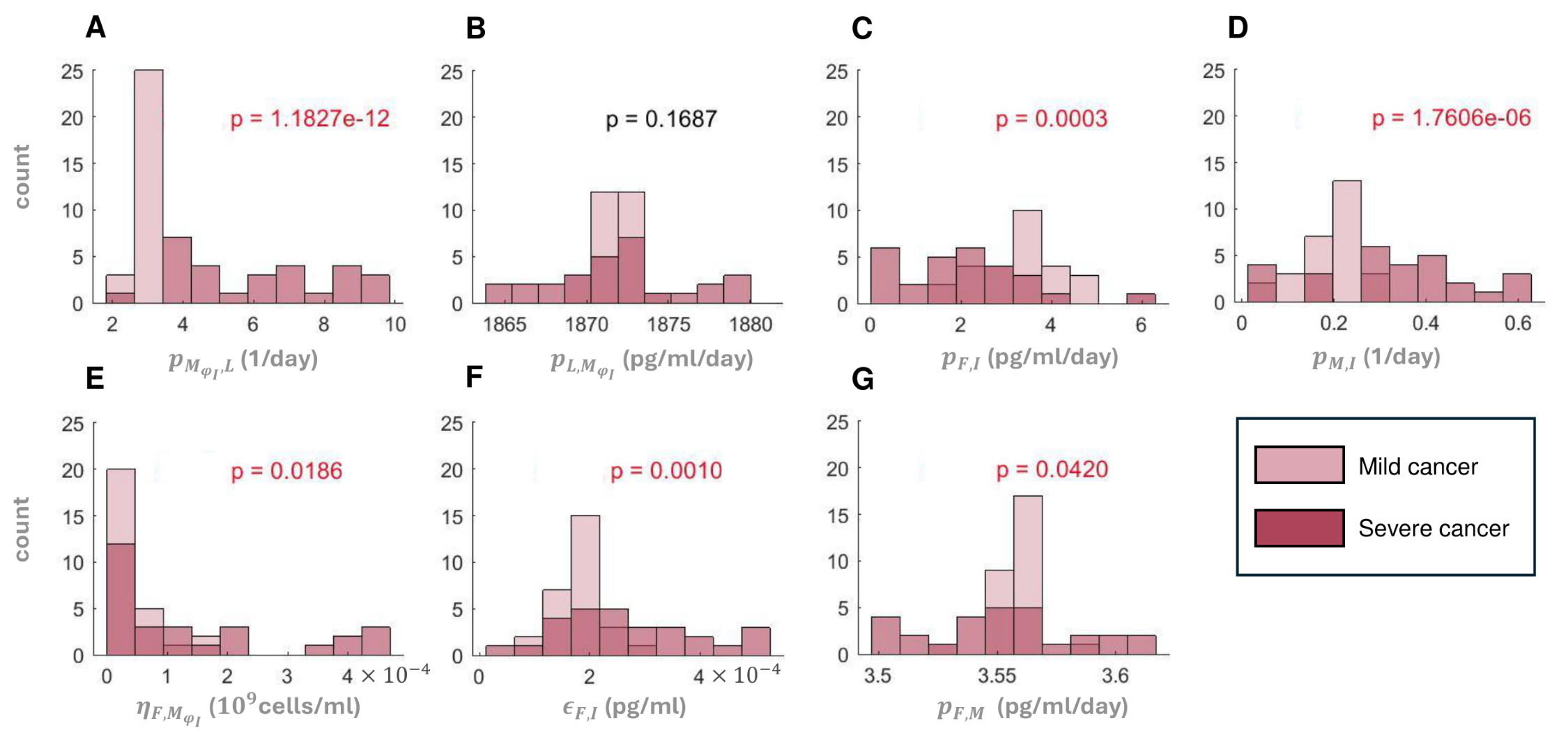

Supplement: S7 Fig — A) Monocyte-to-macrophage differentiation by IL-6, B) IL-6 production by inflammatory macrophages, C) IFN production rates by infected cells, D) Monocyte recruitment by infected cells, E) EC50 concentration of inflammatory macrophages on the IFN production, F) Cell-related IC50 concentration of IFN on virus production, and G) IFN production by monocytes. Statistically significant differences were found for pMϕI,L, pF,I, pM,I, ηF,MϕI, ∈F,I, and pF,M. Red p-values indicate statistically significant differences in distributions (p < 0.05). (TIF) [file pcbi.1013170.s007.tif]

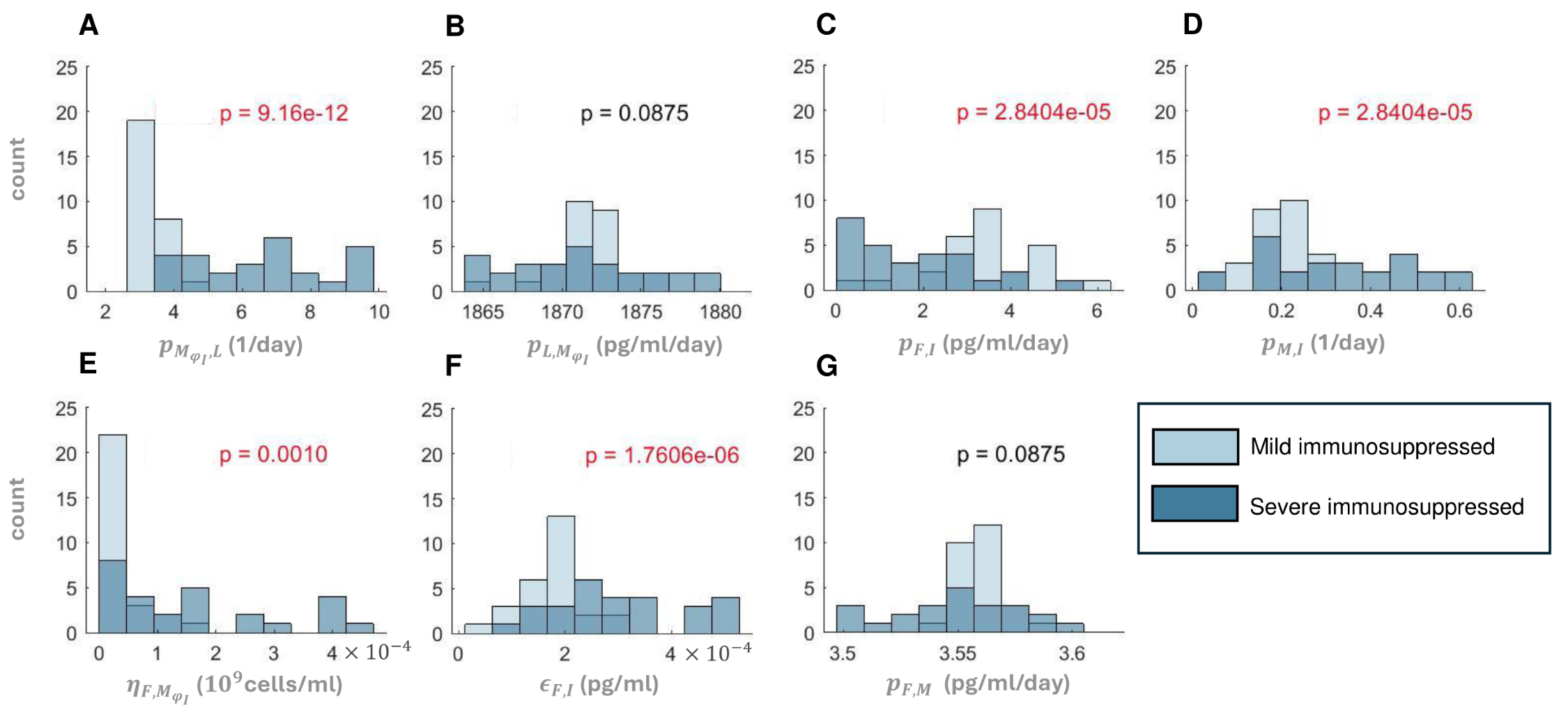

Supplement: S8 Fig — A) Monocyte-to-macrophage differentiation by IL-6, B) IL-6 production by inflammatory macrophages, C) IFN production rates by infected cells, D) Monocyte recruitment by infected cells, E) EC50 concentration of inflammatory macrophages on the IFN production, F) Cell-related IC50 concentration of IFN on virus production, and G) IFN production by monocytes. Statistically significant differences were found for pMϕI,L, pF,I, pM,I, ηF,MϕI, and∈F,I. Red p-values indicate statistically significant differences in distributions (p < 0.05). (TIF) [file pcbi.1013170.s008.tif]

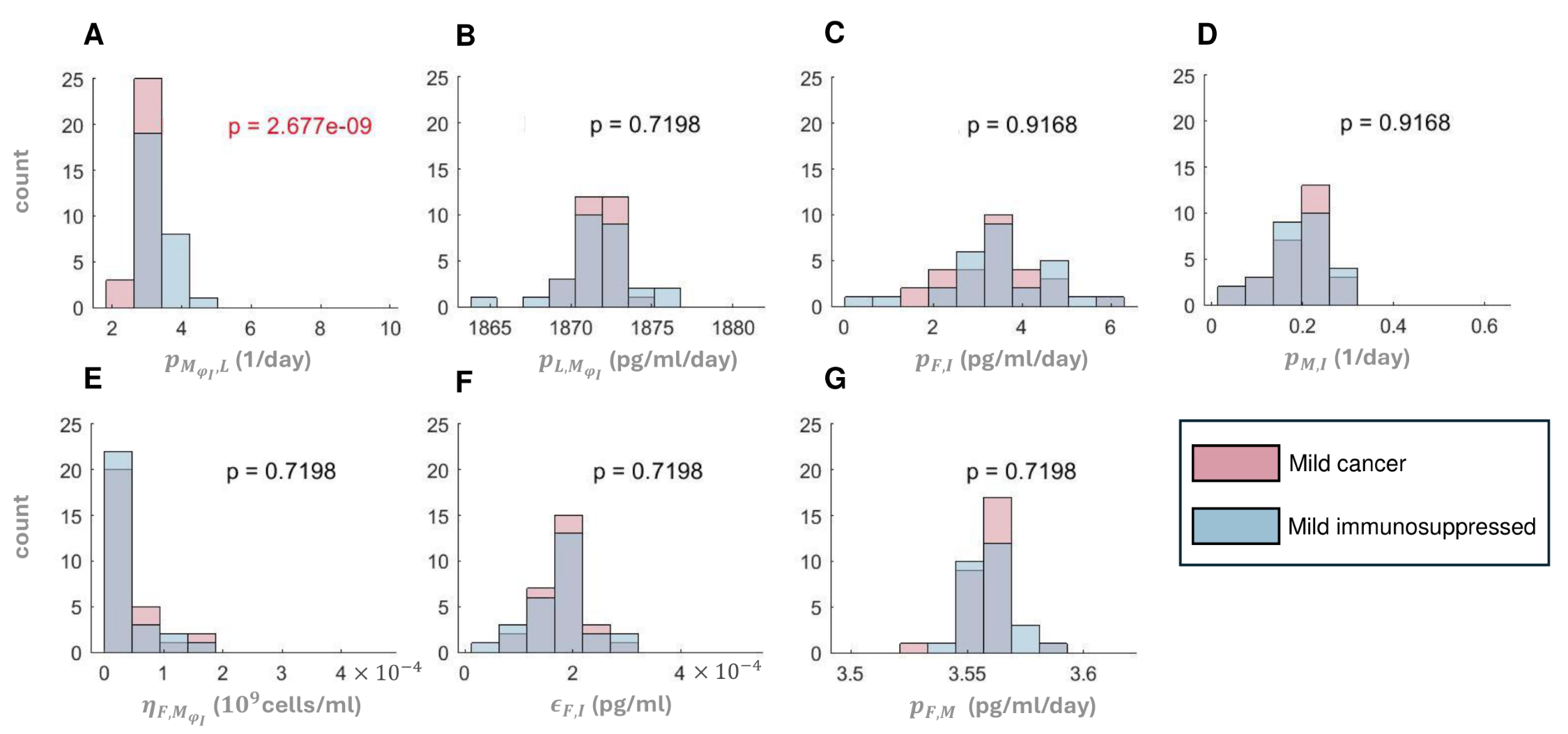

Supplement: S9 Fig — Rate of A) monocyte-to-macrophage differentiation by IL-6, B) IL-6 production by inflammatory macrophages, C) IFN production by infected cells, D) monocyte recruitment by infected cells, E) EC50 concentration of inflammatory macrophages on the IFN production, F) cell-related IC50 concentration of IFN on virus production, and G) IFN production by monocytes. Statistically significant differences were found for pMϕI,L. Red p-values indicate statistically significant differences in distributions (p < 0.05). (TIF) [file pcbi.1013170.s009.tif]

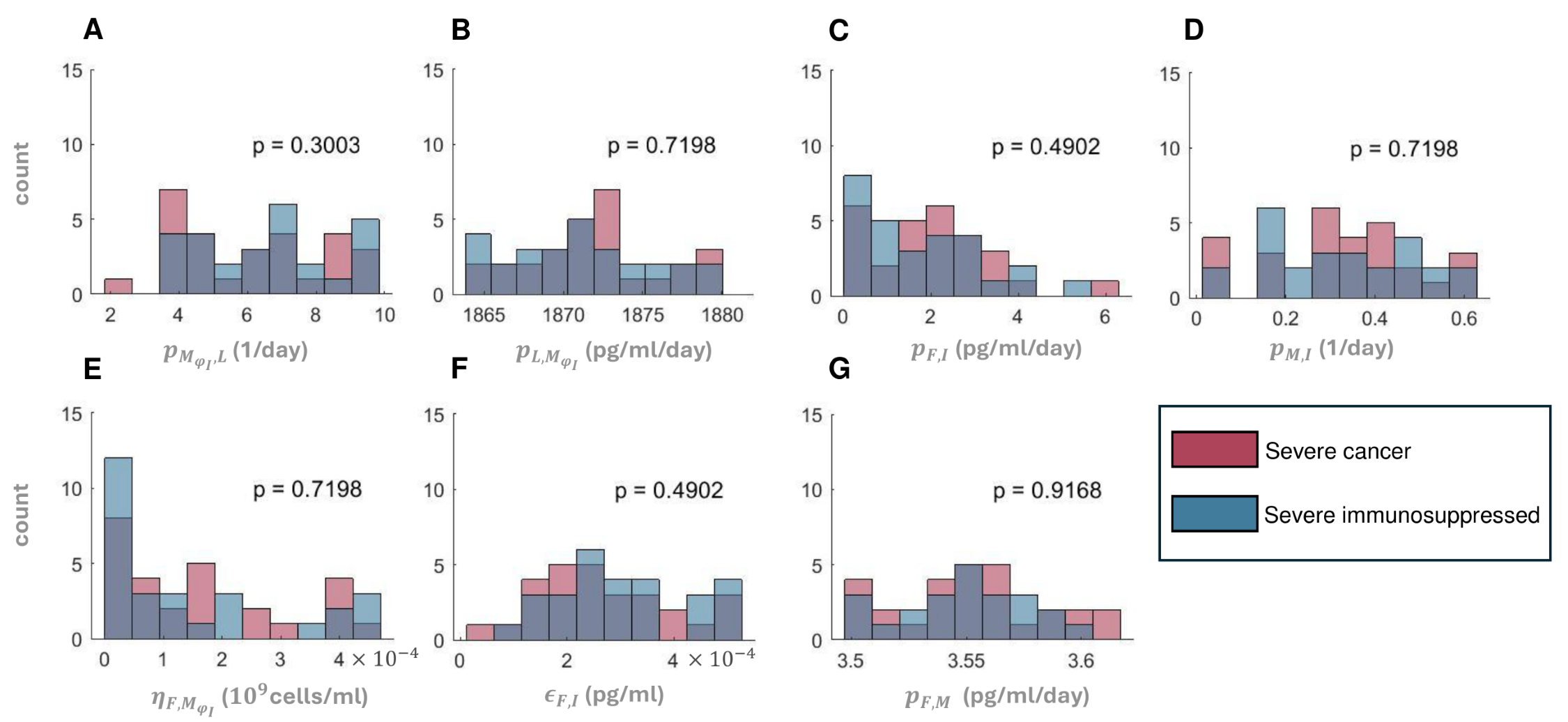

Supplement: S10 Fig — Rate of A) monocyte-to-macrophage differentiation by IL-6, B) IL-6 production by inflammatory macrophages, C) IFN production by infected cells, D) monocyte recruitment by infected cells, E) EC50 concentration of inflammatory macrophages on the IFN production, F) cell-related IC50 concentration of IFN on virus production, and G) IFN production by monocytes. Statistically significant differences were not found in any of the parameters. Red p-values indicate statistically significant differences in distributions (p < 0.05). (TIF) [file pcbi.1013170.s010.tif]

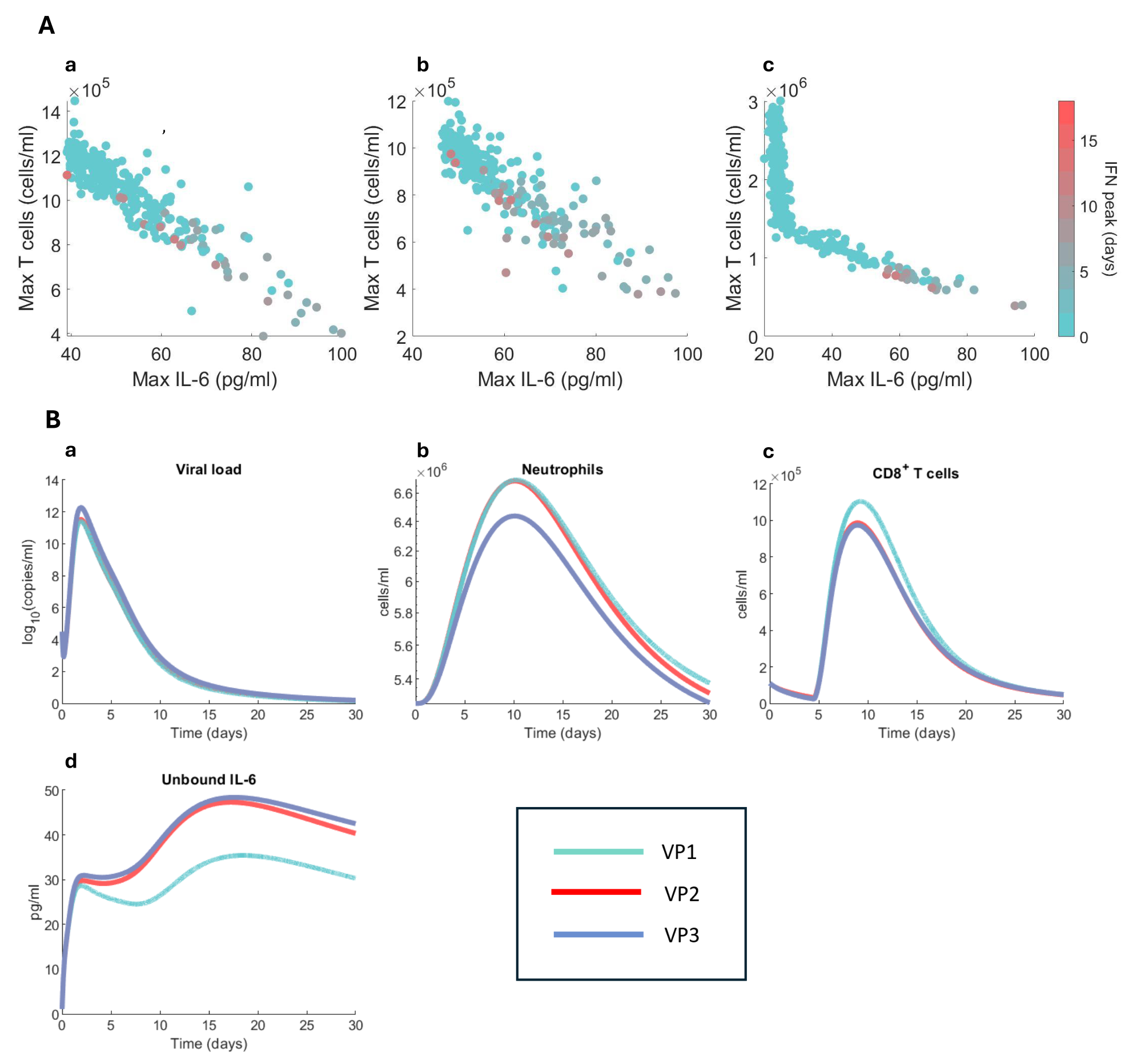

Supplement: S11 Fig — (A) Correlation between maximum T cell concentrations and maximum IL-6 in a) COVID-19 + cancer VPC, b) COVID-19 + immunosuppressed VPC, c) COVID-19 + reference VPC. (B) Model simulation for ‘outlier virtual patients’ (VP1, VP2, and VP3) showing a) viral loads, b) neutrophil concentrations, c) CD8 + T cells, and d) IL-6 concentrations. (TIF) [file pcbi.1013170.s011.tif]

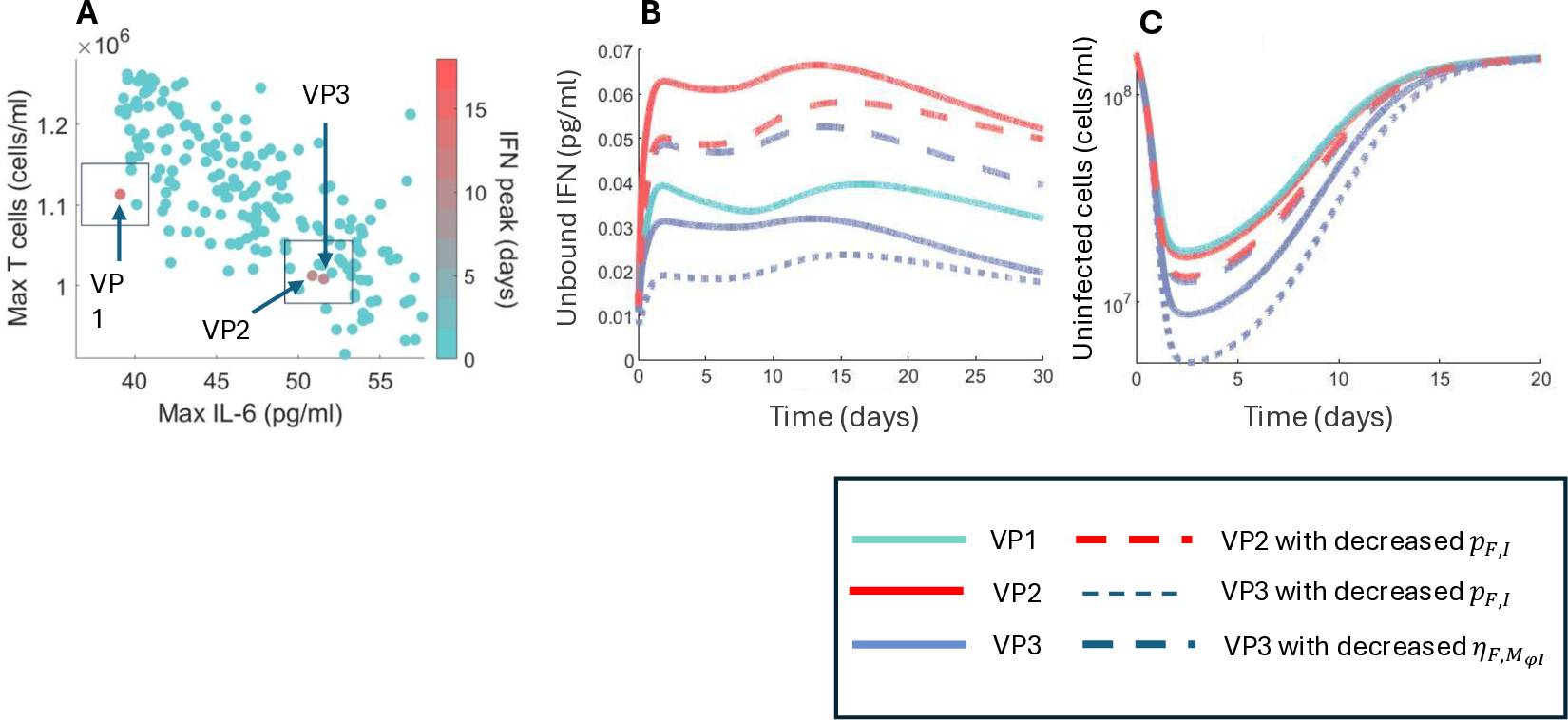

Supplement: S12 Fig — A) Relationships between maximum IL-6, maximum T cell, and peak IFN concentrations in VP1, VP2, and VP3 in the COVID-19 + cancer VPC. B) Unbound IFN dynamics before and after decreasing values of parameters associated with IFN production. C) Uninfected cell dynamics of VP2 and VP3 before and after decreasing values of parameters associated with IFN production. (TIF) [file pcbi.1013170.s012.tif]

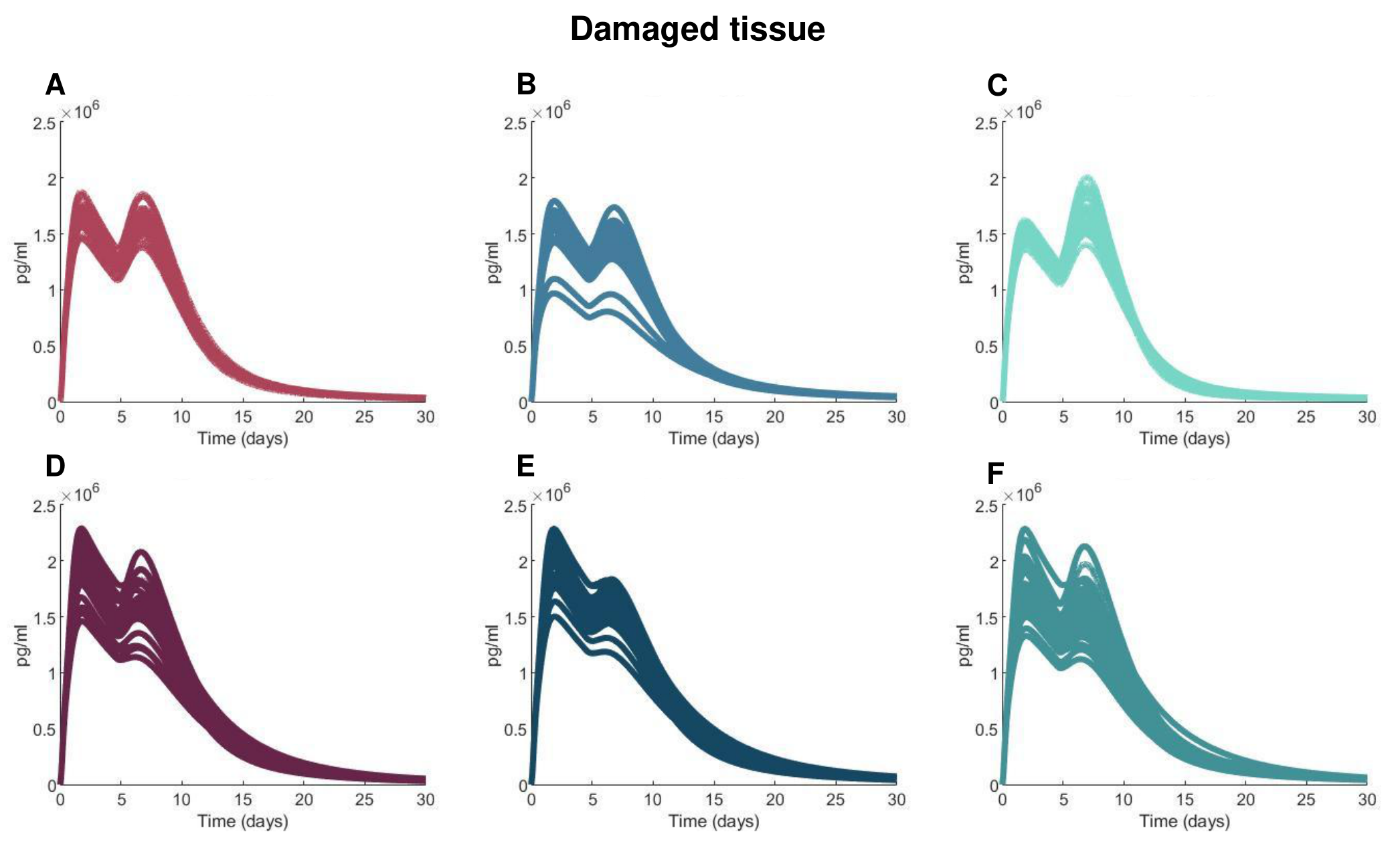

Supplement: S13 Fig — Dynamics of damaged tissue in A) mild COVID-19 + cancer patients, B) mild COVID-19 + immunosuppressed patients, C) mild COVID-19 + reference patients, D) severe COVID-19 + cancer patients, E) severe COVID-19 + immunosuppressed patients, F) severe COVID-19 + reference patients. (TIF) [file pcbi.1013170.s013.tif]

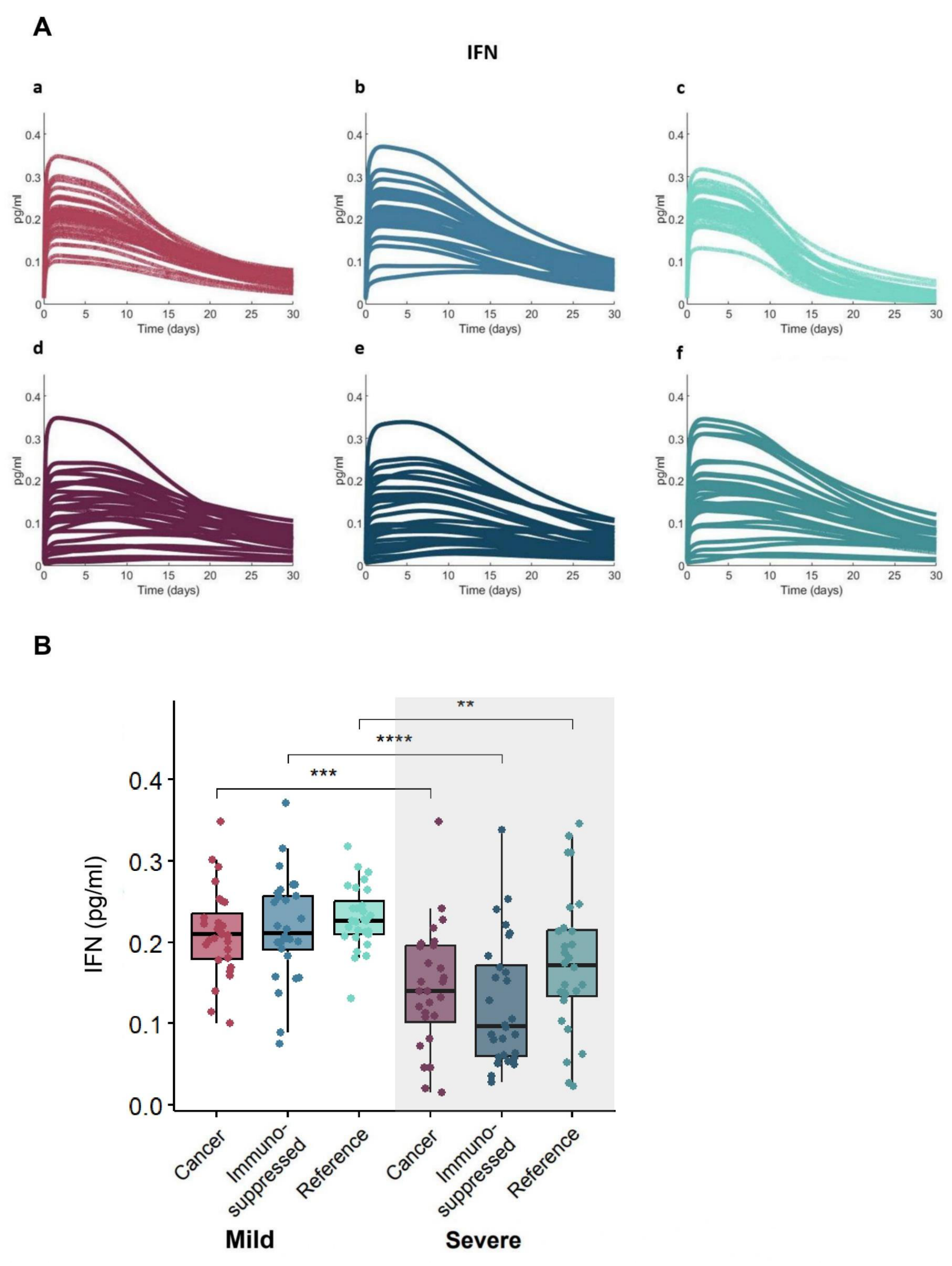

Supplement: S14 Fig — Dynamics of IFN in A: a) Mild COVID-19 + cancer patients, b) Mild COVID-19 + immunosuppressed patients, c) mild COVID-19 + reference patients, d) Severe COVID-19 + cancer patients, e) Severe COVID-19 + immunosuppressed patients, f) Severe COVID-19 + reference patients. B) Model predictions of mean values of maximal IFN concentrations in mild and severe virtual patients. Statistical differences in maximal IFN values between groups are marked by a level of significance (*) estimated based on p-values above the box plots (*, p < 0.05; **, p < 0.005; ***, p < 0.0005; ****, p < 5e-5). A pairwise non-parametric Wilcoxon test was used to assess statistical significance (see Methods). (TIF) [file pcbi.1013170.s014.tif]
