# Supplementary material for: Using virtual patient cohorts to uncover immune response differences in cancer and immunosuppressed COVID-19 patients
Source: PLoS Comput Biol. 2025 Jun 9;21(6):e1013170. doi: 10.1371/journal.pcbi.1013170 (PMC12180667; doi:10.1371/journal.pcbi.1013170)
Supplement: S1 Text — Viral kinetic parameters. Table B. Cell production, recruitment, and activation rates. Table C. Cell-related half-effect (EC50), IC50, and Hill coefficient (h) parameters. Table D. Cell- and virus-induced death rates. Table E. Cell death and virus decay rates. Table F. Cytokine production rates. Table G. Cytokine production regulation parameters. Table H. Cytokine linear clearance and internalization rates. Table I. Cytokine binding/unbinding rates and stoichiometric constants. Table J. Number of cellular receptors and cytokine molecular weights. Table K. Initial conditions. Table L. List of variables in the model equations. Table M. Parameter values of outlier virtual patients. Key immune response parameters were adjusted to analyze their impact on severity. (DOCX) [file pcbi.1013170.s015.docx]

**Supplementary INFORMATION to: Using virtual patient cohorts to uncover differences in immune responses to COVID-19 in cancer and immunosuppressed patients**

Sonia T. Gazeau, Xiaoyan Deng, Elsa Brunet-Ratnasingham, Daniel Kaufmann, Catherine Larochelle, Penelope A. Morel, Jane M. Heffernan, Courtney L. Davis, Amber M. Smith, Adrianne L. Jenner, Morgan Craig

Correspondence to: morgan.craig@umontreal.ca

In our work, we applied a mathematical model describing the immune response against SARS-CoV-2 from Jenner et al. [1] consisting of 17 differential equations (see below). Model schematics and further descriptions are provided in the Main Text. The model parameter values defining the reference virtual patient cohort (VPC) and initial conditions were taken directly from Jenner et al. [1]. Briefly, initial cytokine and cell concentrations were taken or estimated based on the literature (Table K). Cytokine molecular weights and the number of corresponding receptors on the binding cell were taken from prior work (Table J). Cytokine binding and unbinding rates were estimated based on these values (Table I). Cytokine clearance and internalization rates (Table H) were calculated assuming exponential clearance and the usual half-life relationship, i.e., $k_{lin}=ln2/t_{1/2}$ where $k_{lin}$is the clearance rate and $t_{1/2}$ is the cytokine half-life. The rate of IL-6 internalization ($k_{int_{L}})$ was estimated by fitting the model to IL-6 data, with the remaining parameters taken from the literature as indicated in Table H. The monocyte and neutrophil reservoir dynamics are presented in Table L and the corresponding parameters in Table B. Parameters related to CD8^+^ T cell recruitment and expansion can be found in Table B, Table C and Table A (delay in CD8+ T cells arrival). The cell death rates are presented in Table D and Table E. The remaining parameters were obtained by fitting to the variety of data as described in the Supplementary Information from Jenner et al. [1] or were estimated in the absence of infection to ensure the model maintained homeostasis (Table K). For full details, we refer readers to the Supplementary Information from Jenner et al. [1]

**Model equations**

$$\frac{dV}{dt}=pI-\delta_{V,M\Phi}M_{\Phi I}V-\delta_{V,N}NV-d_{V}V,$$

$$\frac{dS}{dt}=\lambda_{S}\left( 1-\frac{S+I+R+D}{S_{max}} \right)S-\beta SV-\frac{\rho\delta_{N}SN^{h_{N}}}{N^{h_{N}}+IC_{50,N}^{h_{N}}},$$

$$\frac{dI}{dt}=\frac{\beta\epsilon_{F,I}}{F_{B}+\epsilon_{F,I}}S\left( t-\tau_{I} \right)V\left( t-\tau_{I} \right)-d_{I}I-\frac{\delta_{N}IN^{h_{N}}}{N^{h_{N}}+IC_{50,N}^{h_{N}}}-\delta_{I,M\Phi}M_{\Phi I}I-\delta_{I,T}TI,$$

$$\frac{dR}{dt}=\lambda_{S}\left( 1-\frac{S+I+R+D}{S_{max}} \right)R+\frac{\beta F_{B}}{F_{B}+\epsilon_{F,I}}SV-\frac{\rho\delta_{N}RN^{h_{N}}}{N^{h_{N}}+IC_{50,N}^{h_{N}}},$$

$$\frac{dD}{dt}=d_{I}I+\frac{\delta_{N}\left( \rho S+\rho R+I \right)N^{h_{N}}}{N^{h_{N}}+IC_{50,N}^{h_{N}}}+\delta_{I,M\Phi}\left( M_{\Phi I} \right)I+ \delta_{I,T}TI-d_{D}D+\delta_{M\Phi,D}M_{\Phi I} -\delta_{D,M\Phi}M_{\Phi R}D-\delta_{D,M\Phi}M_{\Phi I}D,$$

$$\frac{dM_{\Phi R}}{dt}=-a_{I,M\Phi}M_{\Phi R}\left( I+D \right)+\left( 1-\frac{M_{\Phi R}}{M_{\Phi max}} \right)\frac{\lambda_{M_{\Phi}}M_{\Phi I}}{V+\epsilon_{V,M\Phi}}-d_{M_{\Phi R}}M_{\Phi R},$$

$$\frac{dM_{\Phi I}}{dt}=a_{I,M\Phi}M_{\Phi R}\left( I+D \right)+\frac{p_{M_{\Phi I,G}}G_{B}^{h_{M,M\Phi}}M}{G_{B}^{h_{M,M\Phi}}+\epsilon_{G,M_{\Phi I}}^{h_{M,M\Phi}}}+\frac{p_{M_{\Phi I,L}}L_{B}M}{L_{B}+\epsilon_{L,M_{\Phi}}}-d_{M_{\Phi I}}M_{\Phi I}-\delta_{M_{\Phi},D}DM_{\Phi I}-\left( 1-\frac{M_{\Phi R}}{M_{\Phi max}} \right)\frac{\lambda_{M_{\Phi}}M_{\Phi I}}{V+\epsilon_{V,M_{\Phi}}},$$

$$\frac{dM}{dt}=\left( M_{prod}^{*}+\left( \psi_{M}^{max}-M_{prod}^{*} \right)\frac{G_{B}^{h_{M}}}{G_{B}^{h_{M}}+ \epsilon_{G,M}^{h_{M}}} \right)M_{R}+\frac{p_{M,I}IM}{I+\epsilon_{I,M}}-\frac{p_{M_{\Phi I},G}G_{B}^{h_{M,M\Phi}}M}{G_{B}^{h_{M,M\Phi}}+\epsilon_{G,M_{\Phi I}}^{h_{M,M\Phi}}}-\frac{p_{M_{\Phi I},L}L_{B}M}{L_{B}+\epsilon_{L,M_{\Phi}}}-d_{M}M,$$

$$\frac{dN}{dt}=\left( N_{prod}^{*}+\left( \psi_{N}^{max}-N_{prod}^{*} \right)\frac{C_{BF}-C_{BF}^{*}}{C_{BF}-C_{BF}^{*}+\epsilon_{C,N}} \right)N_{R}+\frac{p_{N,L}L_{B}}{L_{B}+\epsilon_{L,N}}-d_{N}N,$$

$$\frac{dT}{dt}=\frac{p_{T,I}I\left( t-\tau_{T} \right)\epsilon_{L.T}}{L_{B}+\epsilon_{L,T}}+\frac{p_{T,F}F_{B}T}{F_{B}+\epsilon_{F,T}}-d_{T}T,$$

$$\frac{dL_{U}}{dt}=\frac{p_{L.I}I}{I+\eta_{L,I}}+\frac{p_{L,M_{\Phi I}}M_{\Phi I}}{M_{\Phi I}+\eta_{L,M_{\Phi I}}}+\frac{p_{L,M}M}{M+\eta_{L,M}}-k_{lin_{L}}L_{U}-k_{B_{L}}\left( \left( M+N+T \right)A_{L}-L_{B} \right)L_{U}+k_{U_{L}}L_{B},$$

$$\frac{dL_{B}}{dt}=-k_{int_{L}}L_{B}+k_{B_{L}}\left( \left( M+N+T \right)A_{L}-L_{B} \right)L_{U}-k_{U_{L}}L_{B},$$

$$\frac{dG_{U}}{dt}=\frac{p_{G,M_{\Phi I}}M_{\Phi I}}{M_{\Phi I}+\eta_{G,M_{\Phi}}}+\frac{p_{G.M}M}{M+\eta_{G,M}}-k_{lin_{G}}G_{U}-k_{B_{G}}\left( MA_{G}-G_{B} \right)G_{U}+k_{U_{G}G_{B}},$$

$$\frac{dG_{B}}{dt}=-k_{int_{G}}G_{B}+k_{B_{G}}\left( MA_{G}-G_{B} \right)G_{U}-k_{U_{G}}G_{B},$$

$$\frac{dC_{U}}{dt}=\frac{p_{C,M}M}{M+\eta_{C,M}}-k_{lin_{C}}C_{U}-k_{B_{C}}\left( NA_{C}-C_{B} \right)\left( C_{U} \right)^{POW}+k_{U_{C}}C_{B},$$

$$\frac{dC_{B}}{dt}=-k_{int_{C}}C_{B}+k_{B_{C}}\left( NA_{C}-C_{B} \right)\left( C_{U} \right)^{POW}-k_{U_{C}}C_{B},$$

$$\frac{dF_{B}}{dt}=-{k_{int}}_{F}F_{B}+k_{B_{F}}\left( \left( T+I \right)A_{F}-F_{B} \right)F_{U}-k_{U_{F}}F_{B},$$

where

$$A_{L}=\frac{MM_{L}}{6.02214\times{10}^{23}}\left( K_{L,N}+K_{L,T}+K_{L,M} \right)\cdot\left( \frac{{10}^{-3}}{5000} \right),$$

$$A_{G}=\frac{MM_{G}}{6.02214\times{10}^{23}}K_{G,M}\cdot\left( \frac{{10}^{-3}}{5000} \right),$$

$$A_{C}=\hat{p}\frac{MM_{C}}{6.02214\times{10}^{23}}K_{C,N}\cdot\left( \frac{{10}^{1}}{5000} \right),$$

$$A_{F}=\frac{MM_{F}}{6.02214\times{10}^{23}}\left( K_{F,T}+K_{F,I} \right)\cdot\left( \frac{{10}^{-3}}{5000} \right).$$

**Parameter Values**

| **Parameter** | **Description** | **Value** | **Units** |
| --- | --- | --- | --- |
| $p$ | Lytic viral production rate | 2.59 | log(copies/ml)/${10}^{9}$cells/day |
| $\lambda_{S}$ | Proliferation of epithelial cells | 0.74 | 1/day |
| $S_{max}$ | Epithelial cells carrying capacity | $S_{0}$ | ${10}^{9}$ cells |
| $\lambda_{M\Phi}$ | Production of alveolar macrophages | 5943 | log(cop/ml)/day |
| $M_{\Phi max}$ | Alveolar macrophage carrying capacity | $M_{\Phi R,0}$ | ${10}^{9}$ cells/ml |
| $\beta$ | SARS-CoV-2 virus infection rate | 0.29 | 1/log(copies/ml)/day |
| $\tau_{I}$ | Eclipse time | 0.17 | day |
| $d_{I}$ | Death rate of infected cells | 0.1 | 1/day |
| $\tau_{T}$ | Delay in CD8+ T cells arrival [2] | 4.5 | 1/day |

**Table A. Viral kinetic parameters.**

| **Parameter** | **Description** | **Value** | **Units** |
| --- | --- | --- | --- |
| $p_{M_{\Phi I},G}$ | Monocyte-to-macrophage differentiation  by GM-CSF [3] | 1.7 | 1/day |
| $p_{M_{\Phi I},L}$ | Monocyte-to-macrophage differentiation by IL-6 [3] | 1.7 | 1/day |
| $a_{I,M\Phi}$ | Activation of macrophages by infected and dead cells$ADDIN ZOTERO\_ITEM CSL\_CITATION \{"citationID":"sj0S2Bso","properties":\{"formattedCitation":"[4,5]","plainCitation":"[4,5]","noteIndex":0\},"citationItems":[\{"id":"I8kjLVwa/bYZfl1Av","uris":["http://zotero.org/users/local/2x6NG6vE/items/9CMP7KLM"],"itemData":\{"id":442,"type":"article-journal","container-title":"Bulletin of Mathematical Biology","DOI":"10.1007/s11538-017-0315-0","ISSN":"0092-8240, 1522-9602","issue":"9","journalAbbreviation":"Bull Math Biol","language":"en","page":"1979-1998","source":"DOI.org (Crossref)","title":"A Mathematical Model for the Macrophage Response to Respiratory Viral Infection in Normal and Asthmatic Conditions","volume":"79","author":[\{"family":"Lee","given":"Junehyuk"\},\{"family":"Adler","given":"Frederick R."\},\{"family":"Kim","given":"Peter S."\}],"issued":\{"date-parts":[["2017",9]]\}\}\},\{"id":"I8kjLVwa/bsmkwwv7","uris":["http://zotero.org/users/local/2x6NG6vE/items/7QSC5M72"],"itemData":\{"id":440,"type":"article-journal","abstract":"Sustained-release delivery systems, such as hydrogels, significantly improve cancer therapies by extending the treatment efficacy and avoiding excess wash-out. Combined virotherapy and immunotherapy (viro-immunotherapy) is naturally improved by these sustained-release systems, as it relies on the continual stimulation of the antitumour immune response. In this article, we consider a previously developed viro-immunotherapy treatment where oncolytic viruses that are genetically engineered to infect and lyse cancer cells are loaded onto hydrogels with immature dendritic cells (DCs). The time-dependent release of virus and immune cells results in a prolonged cancer cell killing from both the virus and activated immune cells. Although effective, a major challenge is optimising the release profile of the virus and immature DCs from the gel so as to obtain a minimum tumour size. Using a system of ordinary differential equations calibrated to experimental results, we undertake a novel numerical investigation of different gel-release profiles to determine the optimal release profile for this viro-immunotherapy. Using a data-calibrated mathematical model, we show that if the virus is released rapidly within the first few days and the DCs are released for two weeks, the tumour burden can be significantly decreased. We then find the true optimal gel-release kinetics using a genetic algorithm and suggest that complex profiles present unnecessary risk and that a simple linear-release model is optimal. In this work, insight is provided into a fundamental problem in the growing field of sustained-delivery systems using mathematical modelling and analysis.","container-title":"Applied Sciences","DOI":"10.3390/app10082872","ISSN":"2076-3417","issue":"8","journalAbbreviation":"Applied Sciences","language":"en","license":"https://creativecommons.org/licenses/by/4.0/","page":"2872","source":"DOI.org (Crossref)","title":"Optimising Hydrogel Release Profiles for Viro-Immunotherapy Using Oncolytic Adenovirus Expressing IL-12 and GM-CSF with Immature Dendritic Cells","volume":"10","author":[\{"family":"Jenner","given":"Adrianne L."\},\{"family":"Frascoli","given":"Federico"\},\{"family":"Yun","given":"Chae-Ok"\},\{"family":"Kim","given":"Peter S."\}],"issued":\{"date-parts":[["2020",4,21]]\}\}\}],"schema":"https://github.com/citation-style-language/schema/raw/master/csl-citation.json"\}$[4,5] | 1.1$\times{10}^{3}$ | ml/${10}^{9}$cells$/$day |
| $p_{M,I}$ | Monocyte recruitment rate by infected cells [6] | 0.22 | 1/day |
| $p_{T,F}$ | CD8+ T cell production rate by IFN [7] | 4 | 1/day |
| $p_{N,L}$ | Neutrophils recruitment rate by IL-6 | 0.21 | 1/day |
| $p_{T,L}$ | CD8+ T cell recruitment rate by IL-6 | 4 | 1/day |
| $p_{T,I}$ | CD8+ T cell proliferation rate [8] | 1 | 1/day |
| $M_{prod}^{*}$ | Homeostasis reservoir release rate | 0.13 | 1/day |
| $\psi_{M}^{max}$ | Maximal reservoir release rate | 11.55[9] | 1/day |
| $N_{prod}^{*}$ | Homeostasis reservoir release rate | 0.21 | 1/day |
| $\psi_{N}^{max}$ | Maximal reservoir release rate [10] | 4.13 | 1/day |
| $C_{BF}^{*}$ | Homeostasis neutrophil receptor bound fraction$ADDIN ZOTERO\_ITEM CSL\_CITATION \{"citationID":"MZtvZVk0","properties":\{"formattedCitation":"[10]","plainCitation":"[10]","noteIndex":0\},"citationItems":[\{"id":"I8kjLVwa/w16qKZNg","uris":["http://zotero.org/users/local/2x6NG6vE/items/YGY3JL7Q"],"itemData":\{"id":320,"type":"article-journal","abstract":"The choice of chemotherapy regimens is often constrained by the patient’s tolerance to the side eﬀects of chemotherapeutic agents. This dose-limiting issue is a major concern in dose regimen design, which is typically focused on maximising drug beneﬁts. Chemotherapy-induced neutropenia is one of the most prevalent toxic eﬀects patients experience and frequently threatens the eﬃcient use of chemotherapy. In response, granulocyte colony-stimulating factor (G-CSF) is co-administered during chemotherapy to stimulate neutrophil production, increase neutrophil counts, and hopefully avoid neutropenia. Its clinical use is, however, largely dictated by trial and error processes. Based on up-to-date knowledge and rational considerations, we develop here a physiologically-realistic model to mathematically characterise the neutrophil production in the bone marrow which we then integrate with pharmacokinetic and pharmacodynamic (PKPD) models of a chemotherapeutic agent and an exogenous form of G-CSF. In this work, model parameters represent the average values for a general patient and are extracted from the literature or estimated from available data. The dose eﬀect predicted by the model is conﬁrmed through previously published data. Using our model, we were able to determine clinically-relevant dosing regimens that advantageously reduce the number of rhG-CSF administrations compared to original studies while signiﬁcantly improving the neutropenia status. More particularly, we determine that it could be beneﬁcial to delay the ﬁrst administration of rhG-CSF to day seven post chemotherapy and reduce the number of administrations from ten to three or four for a patient undergoing 14-day periodic chemotherapy.","container-title":"Journal of Theoretical Biology","DOI":"10.1016/j.jtbi.2015.08.015","ISSN":"00225193","journalAbbreviation":"Journal of Theoretical Biology","language":"en","page":"77-89","source":"DOI.org (Crossref)","title":"Neutrophil dynamics during concurrent chemotherapy and G-CSF administration: Mathematical modelling guides dose optimisation to minimise neutropenia","title-short":"Neutrophil dynamics during concurrent chemotherapy and G-CSF administration","volume":"385","author":[\{"family":"Craig","given":"Morgan"\},\{"family":"Humphries","given":"Antony R."\},\{"family":"Nekka","given":"Fahima"\},\{"family":"Bélair","given":"Jacques"\},\{"family":"Li","given":"Jun"\},\{"family":"Mackey","given":"Michael C."\}],"issued":\{"date-parts":[["2015",11]]\}\}\}],"schema":"https://github.com/citation-style-language/schema/raw/master/csl-citation.json"\}$[10] | 1.6$\times{10}^{-5}$ | Unitless |

**Table B. Cell production, recruitment, and activation rates.**

| **Parameter** | **Description** | **Value** | **Units** |
| --- | --- | --- | --- |
| $\epsilon_{F,I}$ | Cell-related half-maximal inhibitory (IC50) concentration of IFN on the virus production | 4.7$\times{10}^{-4}$ | pg/ml |
| $\epsilon_{L,M_{\Phi}}$ | Cell-related IC50 concentration of IL-6 on monocytes to macrophage differentiation | 0.011 | pg/ml |
| $\epsilon_{G,M_{\Phi I}}$ | Cell-related IC50 concentration of GM-CSF on monocyte to macrophage differentiation | 0.027 | pg/ml |
| $\epsilon_{G,M}$ | Cell-related IC50 concentration of GM-CSF recruitment of monocytes | 57.2 | pg/ml |
| $\epsilon_{F,T}$ | Cell-related IC50 concentration of IFN production of CD8+ T cells | 0.004 | pg/ml |
| $\epsilon_{C,N}$ | Cell-related IC50 concentration of G-CSF recruitment of neutrophils$ADDIN ZOTERO\_ITEM CSL\_CITATION \{"citationID":"qcjfDMXj","properties":\{"formattedCitation":"[10]","plainCitation":"[10]","noteIndex":0\},"citationItems":[\{"id":"I8kjLVwa/w16qKZNg","uris":["http://zotero.org/users/local/2x6NG6vE/items/YGY3JL7Q"],"itemData":\{"id":320,"type":"article-journal","abstract":"The choice of chemotherapy regimens is often constrained by the patient’s tolerance to the side eﬀects of chemotherapeutic agents. This dose-limiting issue is a major concern in dose regimen design, which is typically focused on maximising drug beneﬁts. Chemotherapy-induced neutropenia is one of the most prevalent toxic eﬀects patients experience and frequently threatens the eﬃcient use of chemotherapy. In response, granulocyte colony-stimulating factor (G-CSF) is co-administered during chemotherapy to stimulate neutrophil production, increase neutrophil counts, and hopefully avoid neutropenia. Its clinical use is, however, largely dictated by trial and error processes. Based on up-to-date knowledge and rational considerations, we develop here a physiologically-realistic model to mathematically characterise the neutrophil production in the bone marrow which we then integrate with pharmacokinetic and pharmacodynamic (PKPD) models of a chemotherapeutic agent and an exogenous form of G-CSF. In this work, model parameters represent the average values for a general patient and are extracted from the literature or estimated from available data. The dose eﬀect predicted by the model is conﬁrmed through previously published data. Using our model, we were able to determine clinically-relevant dosing regimens that advantageously reduce the number of rhG-CSF administrations compared to original studies while signiﬁcantly improving the neutropenia status. More particularly, we determine that it could be beneﬁcial to delay the ﬁrst administration of rhG-CSF to day seven post chemotherapy and reduce the number of administrations from ten to three or four for a patient undergoing 14-day periodic chemotherapy.","container-title":"Journal of Theoretical Biology","DOI":"10.1016/j.jtbi.2015.08.015","ISSN":"00225193","journalAbbreviation":"Journal of Theoretical Biology","language":"en","page":"77-89","source":"DOI.org (Crossref)","title":"Neutrophil dynamics during concurrent chemotherapy and G-CSF administration: Mathematical modelling guides dose optimisation to minimise neutropenia","title-short":"Neutrophil dynamics during concurrent chemotherapy and G-CSF administration","volume":"385","author":[\{"family":"Craig","given":"Morgan"\},\{"family":"Humphries","given":"Antony R."\},\{"family":"Nekka","given":"Fahima"\},\{"family":"Bélair","given":"Jacques"\},\{"family":"Li","given":"Jun"\},\{"family":"Mackey","given":"Michael C."\}],"issued":\{"date-parts":[["2015",11]]\}\}\}],"schema":"https://github.com/citation-style-language/schema/raw/master/csl-citation.json"\}$[10] | 1.89$\times{10}^{-4}$ | Unitless |
| $\epsilon_{L,N}$ | Cell-related IC50 concentration of IL-6 recruitment of neutrophils | 57.2 | pg/ml |
| $\epsilon_{I,M}$ | Cell-related IC50 concentration of infected cell monocyte recruitment | 0.11 | ${10}^{9}$cells/ml |
| $\epsilon_{L,T}$ | Cell-related IC50 concentration of IL-6 production of CD8+ T cells | $3\times{10}^{-4}$ | pg/ml |
| $\epsilon_{V,M_{\Phi}}$ | Cell-related IC50 concentration of viral load for mac replenishing | 2.96 | log(cop/ml) |
| $\epsilon_{T,I}$ | Cell-related IC50 effect concentration of antigen-driven proliferation$ADDIN ZOTERO\_ITEM CSL\_CITATION \{"citationID":"BFI6Lghp","properties":\{"formattedCitation":"[8]","plainCitation":"[8]","noteIndex":0\},"citationItems":[\{"id":"I8kjLVwa/Pepyq8mO","uris":["http://zotero.org/users/local/2x6NG6vE/items/M4CRB547"],"itemData":\{"id":449,"type":"article-journal","abstract":"One of the most challenging tasks in constructing a mathematical model of cancer treatment is the calculation of biological parameters from empirical data. This task becomes increasingly difficult if a model involves several cell populations and treatment modalities. A sophisticated model constructed by de Pillis\backslash n et al.\backslash n ,\backslash n Mixed immunotherapy and chemotherapy of tumours: Modelling, applications and biological interpretations\backslash n , J. Theor. Biol. 238 (2006), pp. 841-862; involves tumour cells, specific and non-specific immune cells (natural killer (NK) cells, CD8\backslash n +\backslash n T cells and other lymphocytes) and employs chemotherapy and two types of immunotherapy (IL-2 supplementation and CD8\backslash n +\backslash n T-cell infusion) as treatment modalities. Despite the overall success of the aforementioned model, the problem of illustrating the effects of IL-2 on a growing tumour remains open. In this paper, we update the model of de Pillis\backslash n et al.\backslash n and then carefully identify appropriate values for the parameters of the new model according to recent empirical data. We determine new NK and tumour antigen-activated CD8\backslash n +\backslash n T-cell count equilibrium values; we complete IL-2 dynamics; and we modify the model in de Pillis\backslash n et al.\backslash n to allow for endogenous IL-2 production, IL-2-stimulated NK cell proliferation and IL-2-dependent CD8\backslash n +\backslash n T-cell self-regulations. Finally, we show that the potential patient-specific efficacy of immunotherapy may be dependent on experimentally determinable parameters.","container-title":"Computational and Mathematical Methods in Medicine","DOI":"10.1080/17486700802216301","ISSN":"1748-670X, 1748-6718","issue":"3","journalAbbreviation":"Computational and Mathematical Methods in Medicine","language":"en","page":"165-184","source":"DOI.org (Crossref)","title":"Mathematical Model Creation for Cancer Chemo-Immunotherapy","volume":"10","author":[\{"family":"De Pillis","given":"Lisette"\},\{"family":"Fister","given":"K. Renee"\},\{"family":"Gu","given":"Weiqing"\},\{"family":"Collins","given":"Craig"\},\{"family":"Daub","given":"Michael"\},\{"family":"Gross","given":"David"\},\{"family":"Moore","given":"James"\},\{"family":"Preskill","given":"Benjamin"\}],"issued":\{"date-parts":[["2009",1]]\}\}\}],"schema":"https://github.com/citation-style-language/schema/raw/master/csl-citation.json"\}$[8] | ${10}^{-6}$ | ${10}^{9}$ cells/ml |
| $h_{M}$ | Hill coefficient of GM-CSF monocyte recruitment | 1.67 | Unitless |
| $h_{M,M_{\Phi}}$ | Hill coefficient of GM-CSF monocyte to macrophages | 2.03 | Unitless |
| $h_{N}$ | Hill coefficient of neutrophil-induced damage | 3.02 | Unitless |
| $IC_{50,N}$ | Neutrophil IC50 concentration of neutrophil-induced damage | 0.047 | ${10}^{9}$ cells/ml |

**Table C. Cell-related half-effect (**$\boldsymbol{\epsilon}$**), IC50 (**$\boldsymbol{I}\boldsymbol{C}_{\boldsymbol{50}}$**), and Hill coefficient (h) parameters**

| **Parameter** | **Description** | **Value** | **Units** |
| --- | --- | --- | --- |
| $\delta_{V,M\Phi}$ | Rate of viral clearance by macrophages | 768 | ml/(${10}^{9}$cells) $\times$ 1/day |
| $\delta_{V,N}$ | Rate of viral clearance by neutrophils | 2304 | ml/(${10}^{9}$cells) $\times$ 1/day |
| $\delta_{N}$ | Rate of neutrophil-inflicted damage | 1.68 | 1/day |
| $\rho$ | Bystander death modulation constant | 0.5 | Unitless |
| $\delta_{I,M\Phi}$ | Rate macrophages phagocytose infected cells [11] | 121 | ml/(${10}^{9}$cells) $\times$ 1/day |
| $\delta_{I,T}$ | Rate CD8+ T cells induce apoptosis in infected cells$ADDIN ZOTERO\_ITEM CSL\_CITATION \{"citationID":"X50Y6qs1","properties":\{"formattedCitation":"[12]","plainCitation":"[12]","noteIndex":0\},"citationItems":[\{"id":"I8kjLVwa/Wz2jEZmP","uris":["http://zotero.org/users/local/2x6NG6vE/items/7K6MRRLZ"],"itemData":\{"id":466,"type":"article-journal","abstract":"ABSTRACT\backslash n The cellular immune response to primary influenza virus infection is complex, involving multiple cell types and anatomical compartments, and is difficult to measure directly. Here we develop a two-compartment model that quantifies the interplay between viral replication and adaptive immunity. The fidelity of the model is demonstrated by accurately confirming the role of CD4 help for antibody persistence and the consequences of immune depletion experiments. The model predicts that drugs to limit viral infection and/or production must be administered within 2 days of infection, with a benefit of combination therapy when administered early, and cytotoxic CD8 T cells in the lung are as effective for viral clearance as neutralizing antibodies when present at the time of challenge. The model can be used to investigate explicit biological scenarios and generate experimentally testable hypotheses. For example, when the adaptive response depends on cellular immune cell priming, regulation of antigen presentation has greater influence on the kinetics of viral clearance than the efficiency of virus neutralization or cellular cytotoxicity. These findings suggest that the modulation of antigen presentation or the number of lung resident cytotoxic cells and the combination drug intervention are strategies to combat highly virulent influenza viruses. We further compared alternative model structures, for example, B-cell activation directly by the virus versus that through professional antigen-presenting cells or dendritic cell licensing of CD8 T cells.","container-title":"Journal of Virology","DOI":"10.1128/JVI.00098-09","ISSN":"0022-538X, 1098-5514","issue":"14","journalAbbreviation":"J Virol","language":"en","page":"7151-7165","source":"DOI.org (Crossref)","title":"Simulation and Prediction of the Adaptive Immune Response to Influenza A Virus Infection","volume":"83","author":[\{"family":"Lee","given":"Ha Youn"\},\{"family":"Topham","given":"David J."\},\{"family":"Park","given":"Sung Yong"\},\{"family":"Hollenbaugh","given":"Joseph"\},\{"family":"Treanor","given":"John"\},\{"family":"Mosmann","given":"Tim R."\},\{"family":"Jin","given":"Xia"\},\{"family":"Ward","given":"Brian M."\},\{"family":"Miao","given":"Hongyu"\},\{"family":"Holden-Wiltse","given":"Jeanne"\},\{"family":"Perelson","given":"Alan S."\},\{"family":"Zand","given":"Martin"\},\{"family":"Wu","given":"Hulin"\}],"issued":\{"date-parts":[["2009",7,15]]\}\}\}],"schema":"https://github.com/citation-style-language/schema/raw/master/csl-citation.json"\}$[12] | 238 | ml/(${10}^{9}$cells) $\times$ 1/day |
| $\delta_{M\Phi,D}$ | Rate macrophages die from phagocytosis [13,14] | 6.06 | ml/(${10}^{9}$cells) $\times$ 1/day |
| $\delta_{D,M\Phi}$ | Rate macrophages phagocytose dead cells | 8.03 | ml/(${10}^{9}$cells) $\times$ 1/day |

**Table D. Cell- and virus-induced death rates**

| **Parameter** | **Description** | **Value** | **Units** |
| --- | --- | --- | --- |
| $d_{V}$ | Viral decay rate | 1.81 | 1/day |
| $d_{D}$ | Degradation rate of apoptotic cells [15] | 8 | 1/day |
| $d_{M_{\Phi R}}$ | Alveolar macrophage death rate [16,17] | 0 | 1/day |
| $d_{M_{\Phi I}}$ | Inflammatory macrophage death rate [18] | 0.3 | 1/day |
| $d_{M}$ | Monocyte death rate [19] | 0.76 | 1/day |
| $d_{N}$ | Neutrophil death rate [20] | 1.28 | 1/day |
| $d_{T}$ | CD8+ T cell death rate [21] | 0.4 | 1/day |

**Table E. Cell death and virus decay rates.**

| **Parameter** | **Description** | **Value** | **Units** |
| --- | --- | --- | --- |
| $p_{L,I}$ | Rate of IL-6 production by infected cells | 11.89 | pg/ml/day |
| $p_{{L,M}_{\Phi I}}$ | Rate of IL-6 production by inflammatory macrophages | 1872 | pg/ml/day |
| $p_{L,M}$ | Rate of IL-6 production by monocytes | 72.56 | pg/ml/day |
| $p_{{G,M}_{\Phi I}}$ | Rate of GM-CSF production by inflammatory macrophages | 2626 | pg/ml/day |
| $p_{C,M}$ | Rate of G-CSF production by monocytes | 26.26 | pg/ml/day |
| $p_{G,M}$ | Rate of GM-CSF production by monocytes | 3070 | pg/ml/day |
| $p_{F,I}$ | Rate of IFN production by infected cells | 2.82 | pg/ml/day |
| $p_{{F,M}_{\Phi I}}$ | Rate of IFN production by inflammatory macrophages | 1.3 | pg/ml/day |
| $p_{F,M}$ | Rate of IFN production by monocytes | 3.56 | pg/ml/day |

**Table F. Cytokine production rates.**

| **Parameter** | **Description** | **Value** | **Units** |
| --- | --- | --- | --- |
| $\eta_{L,I}$ | Half-maximal stimulatory (EC50) concentration of infected cells on the IL-6 production | 0.7 | ${10}^{9}$ cells/ml |
| $\eta_{L,M}$ | EC50 concentration of monocytes on the IL-6 production | 0.0045 | ${10}^{9}$ cells/ml |
| $\eta_{L{,M}_{\Phi I}}$ | EC50 concentration of inflammatory macrophages on the IL-6 production | 3.6$\times{10}^{-5}$ | ${10}^{9}$ cells/ml |
| $\eta_{G{,M}_{\Phi I}}$ | EC50 concentration of inflammatory macrophages on the GM-CSF production | 3.6$\times{10}^{-5}$ | ${10}^{9}$ cells/ml |
| $\eta_{G,M}$ | EC50 concentration of monocytes on the GM-CSF production | 0.15 | ${10}^{9}$ cells/ml |
| $\eta_{C,M}$ | EC50 concentration of monocytes on the G-CSF production | 3.05 | ${10}^{9}$ cells/ml |
| $\eta_{F,I}$ | Half-effect concentration of IFN production by infected cells | 0.011 | ${10}^{9}$ cells/ml |
| $\eta_{F{,M}_{\Phi I}}$ | EC50 concentration of inflammatory macrophages on the IFN production | 1.3$\times{10}^{-6}$ | ${10}^{9}$ cells/ml |
| $\eta_{F,M}$ | EC50 concentration of monocytes on the IFN production | 0.54 | ${10}^{9}$ cells/ml |

**Table G. Cytokine production regulation parameters.**

| **Parameter** | **Description** | **Value** | **Units** |
| --- | --- | --- | --- |
| $k_{lin_{L}}$ | Rate of IL-6 renal clearance [22] | 16.6 | 1/day |
| $k_{lin_{G}}$ | Rate of GM-CSF renal clearance [23] | 11.7 | 1/day |
| $k_{lin_{C}}$ | Rate of G-CSF renal clearance [10] | 0.16 | 1/day |
| $k_{lin_{F}}$ | Rate of IFN renal clearance [24] | 18 | 1/day |
| $k_{int_{L}}$ | Internalization rate of IL-6 | 61.8 | 1/day |
| $k_{int_{G}}$ | Internalization rate of GM-CSF [25] | 73.4 | 1/day |
| $k_{int_{C}}$ | Internalization rate of G-CSF [10,25] | 462 | 1/day |
| $k_{int_{F}}$ | Internalization rate of IFN [24,26] | 17 | 1/day |

**Table H. Cytokine linear (renal) clearance and internalization rates.**

| **Parameter** | **Description** | **Value** | **Units** |
| --- | --- | --- | --- |
| $k_{B_{L}}$ | IL-6 binding rate [27] | 0.0018 | ml/pg/day |
| $k_{B_{G}}$ | GM-CSF binding rate [25] | 0.0021 | ml/pg/day |
| $k_{B_{C}}$ | G-CSF binding rate | 2.24 | ml/pg/day |
| $k_{B_{F}}$ | IFN binding rate [26] | 0.011 | ml/pg/day |
| $k_{U_{L}}$ | IL-6 unbinding rate [27] | 22.3 | 1/day |
| $k_{U_{G}}$ | GM-CSF unbinding rate [25] | 522 | 1/day |
| $k_{U_{C}}$ | G-CSF unbinding rate [10] | 184 | 1/day |
| $k_{U_{F}}$ | IFN unbinding rate | 6.07 | 1/day |
| *POW* | Stoichiometric constant (G-CSF)  Stoichiometric constant (IL-6, GM-CSF, IFN) | 1.4608  1 | Unitless |
| $\hat{p}$ | Stoichiometric constant (G-CSF)  Stoichiometric constant (IL-6, GM-CSF, IFN) | 2  1 | Unitless |

**Table I. Cytokine binding/unbinding rates and stoichiometric constant.**

| **Parameter** | **Description** | **Value** | **Units** |
| --- | --- | --- | --- |
| $K_{L,N}$ | No. IL-6 receptors on neutrophils [28] | 720 | sites/cell |
| $K_{L,T}$ | No. IL-6 receptors on T cells [29] | 300 | sites/cell |
| $K_{L,M}$ | No. of IL-6 receptors on monocytes [30] | 509 | sites/cell |
| $K_{G,M}$ | No. of GM-CSF receptors on monocyte [31] | 1058 | sites/cell |
| $K_{C,N}$ | No. of G-CSF receptors on neutrophil [32] | 600 | sites/cell |
| $K_{F,T}$ | No. of IFN receptors on T cells [33] | 1000 | sites/cell |
| $K_{F,I}$ | No. of IF receptors on infected cells [34] | 1300 | sites/cell |
| $MM_{L}$ | Molecular weight of IL-6 [35] | 21000 | g/mol |
| $MM_{G}$ | Molecular weight of GM-CSF [36] | 14000 | g/mol |
| $MM_{C}$ | Molecular weight of G-CSF [10] | 19600 | g/mol |
| $MM_{F}$ | Molecular weight of IFN-$\beta$[37] | 19000 | g/mol |

**Table J. Number of cellular receptors and cytokine molecular weights.**

| **Parameter** | **Description** | **Value** | **Units** |
| --- | --- | --- | --- |
| $V_{0}$ | Initial viral load | 4.5 | log(copies/ml) |
| $S_{0}$ | Initial susceptible cells [38,39] | 0.16 | ${10}^{9}$cells/ml |
| $I_{0}$ | Initial infected cells | 0 | ${10}^{9}$cells/ml |
| $R_{0}$ | Initial resistant cells | 0 | ${10}^{9}$cells/ml |
| $M_{\Phi R,0}$ | Initial resident macrophages$ADDIN ZOTERO\_ITEM CSL\_CITATION \{"citationID":"tLA2Abi7","properties":\{"formattedCitation":"[39]","plainCitation":"[39]","noteIndex":0\},"citationItems":[\{"id":"I8kjLVwa/dFMQjK8b","uris":["http://zotero.org/users/local/2x6NG6vE/items/ZCNAWFRY"],"itemData":\{"id":405,"type":"article-journal","container-title":"126, 332-337","issue":"126, 332-337","journalAbbreviation":"Am. Rev. Respir. Dis.","title":"Cell number and cell characteristics of the normal human lung.","author":[\{"family":"Crapo, J. D., Barry, B. E., Gehr, P., Bachofen, M \& Weibel, E. R.","given":""\}],"issued":\{"date-parts":[["1982"]]\}\}\}],"schema":"https://github.com/citation-style-language/schema/raw/master/csl-citation.json"\}$[39] | 2.7$\times{10}^{-5}$ | ${10}^{9}$cells/ml |
| $M_{\Phi I,0}$ | Initial inflammatory macrophages | 2.9$\times{10}^{-5}$ | ${10}^{9}$cells/ml |
| $M_{0}$ | Initial monocytes [40,41] | 0.0004 | ${10}^{9}$cells/ml |
| $M_{R}$ | Initial reservoir monocytes | 0.0023 | ${10}^{9}$cells/ml |
| $N_{0}$ | Initial neutrophils [10] | 0.0053 | ${10}^{9}$ cells/ml |
| $N_{R}$ | Initial reservoir neutrophils | 0.0316 | ${10}^{9}$cells/ml |
| $T_{0}$ | Initial CD8+ T cells$ADDIN ZOTERO\_ITEM CSL\_CITATION \{"citationID":"7x996xCO","properties":\{"formattedCitation":"[42\backslash\backslash uc0\backslash\backslash u8211\{\}44]","plainCitation":"[42-44]","noteIndex":0\},"citationItems":[\{"id":"I8kjLVwa/eX8Cj7ma","uris":["http://zotero.org/users/local/2x6NG6vE/items/EPVBC2SR"],"itemData":\{"id":411,"type":"article-journal","abstract":"T cell responses to viruses are initiated and maintained in tissue sites; however, knowledge of human antiviral T cells is largely derived from blood. Cytomegalovirus (CMV) persists in most humans, requires T cell immunity to control, yet tissue immune responses remain undefined. Here, we investigated human CMV-specific T cells, virus persistence and CMV-associated T cell homeostasis in blood, lymphoid, mucosal and secretory tissues of 44 CMV seropositive and 28 seronegative donors. CMV-specific T cells were maintained in distinct distribution patterns, highest in blood, bone marrow (BM), or lymph nodes (LN), with the frequency and function in blood distinct from tissues. CMV genomes were detected predominantly in lung and also in spleen, BM, blood and LN. High frequencies of activated CMV-specific T cells were found in blood and BM samples with low virus detection, whereas in lung, CMV-specific T cells were present along with detectable virus. In LNs, CMV-specific T cells exhibited quiescent phenotypes independent of virus. Overall, T cell differentiation was enhanced in sites of viral persistence with age. Together, our results suggest tissue T cell reservoirs for CMV control shaped by both viral and tissue-intrinsic factors, with global effects on homeostasis of tissue T cells over the lifespan.","container-title":"Journal of Experimental Medicine","DOI":"10.1084/jem.20160758","ISSN":"0022-1007, 1540-9538","issue":"3","language":"en","page":"651-667","source":"DOI.org (Crossref)","title":"Tissue reservoirs of antiviral T cell immunity in persistent human CMV infection","volume":"214","author":[\{"family":"Gordon","given":"Claire L."\},\{"family":"Miron","given":"Michelle"\},\{"family":"Thome","given":"Joseph J.C."\},\{"family":"Matsuoka","given":"Nobuhide"\},\{"family":"Weiner","given":"Joshua"\},\{"family":"Rak","given":"Michael A."\},\{"family":"Igarashi","given":"Suzu"\},\{"family":"Granot","given":"Tomer"\},\{"family":"Lerner","given":"Harvey"\},\{"family":"Goodrum","given":"Felicia"\},\{"family":"Farber","given":"Donna L."\}],"issued":\{"date-parts":[["2017",3,6]]\}\}\},\{"id":"I8kjLVwa/j1ZBcBdy","uris":["http://zotero.org/users/local/2x6NG6vE/items/IQF4JSAR"],"itemData":\{"id":413,"type":"article-journal","abstract":"Abstract\backslash n \backslash n Background\backslash n Information on lymphocyte populations (T, B, and natural killer cells) and subpopulations (CD4 and CD8) in India is generally lacking. Measurement of T-cell subsets is important in India for evaluating disease stage and progression in individuals with the human immunodeficiency virus (HIV). Hence, this study was conducted to provide normal ranges of absolute and percentage values of CD4 and CD8 T-lymphocyte subsets and the ratio of CD4 to CD8 in normal Indian adults.\backslash n \backslash n \backslash n Methods\backslash n Flow cytometric analysis (EPICS-XL) was used to determine the range of T-lymphocyte subpopulations in normal Indian blood donors at Command Hospital and the Armed Forces Medical College, Pune, India. The reference population consisted of 94 healthy HIV-seronegative blood donors. T-lymphocyte subsets were analyzed with two-color immunophenotyping of peripheral blood lymphocytes with the use of a lysed whole-blood technique and enumerated.\backslash n \backslash n \backslash n Results\backslash n \backslash n For normal values of various blood components, we found mean values of 2114 cells/\mu l for total lymphocytes, 865 cells/\mu l (40.2\%) for CD4\backslash n +\backslash n lymphocytes, 552 cells/\mu l (31.3\%) for CD8\backslash n +\backslash n lymphocytes, and 1.7 for the CD4:CD8 ratio. The 95\% confidence intervals for the same parameters were 1115-4009 cells/\mu l, 430-1740 cells/\mu l (30.75-49.60\%), 218-1396 cells/\mu l (20.06-42.52\%), and 0.39-3.02 respectively. Females had significantly higher CD4 counts (\backslash n P\backslash n < 0.05), percentage of CD4 lymphocytes (\backslash n P\backslash n < 0.01), and CD4:CD8 ratio (\backslash n P\backslash n < 0.01). Males had a significantly higher percentage of CD8 lymphocytes (\backslash n P\backslash n < 0.01). They also had higher CD8 counts that did not reach significance. Age, ethnicity (Dravidian versus Aryan), smoking, alcohol consumption, and the interval between drawing the blood sample and its analysis were factors that did not produce statistically significant differences in the T-cell subsets studied.\backslash n \backslash n \backslash n \backslash n Conclusions\backslash n When compared with other published series, the CD4 and CD8 values in healthy Indians were no different from those reported in the West. These observations have important clinical implications for the use of T-lymphocyte subset measurements in India, especially in the management of HIV infection. The normal ranges established by this study can be used as a reference for decisions made in clinical practice. Cytometry Part B (Clin. Cytometry) 52B:32-36, 2003. © 2003 Wiley-Liss, Inc.","container-title":"Cytometry Part B: Clinical Cytometry","DOI":"10.1002/cyto.b.10011","ISSN":"1552-4949, 1552-4957","issue":"1","journalAbbreviation":"Cytometry Part B Clinical","language":"en","license":"http://onlinelibrary.wiley.com/termsAndConditions\#vor","page":"32-36","source":"DOI.org (Crossref)","title":"Normal values of CD4 and CD8 lymphocyte subsets in healthy indian adults and the effects of sex, age, ethnicity, and smoking","volume":"52B","author":[\{"family":"Uppal","given":"S. S."\},\{"family":"Verma","given":"Shashi"\},\{"family":"Dhot","given":"P. S."\}],"issued":\{"date-parts":[["2003",3]]\}\}\},\{"id":"I8kjLVwa/bEJr1Dky","uris":["http://zotero.org/users/local/2x6NG6vE/items/QAMLY6S3"],"itemData":\{"id":414,"type":"article-journal","abstract":"Abstract\backslash n \backslash n Only activated and effector memory T cells are thought to access non-lymphoid tissues. In contrast, naive T cells are thought to circulate only between the blood, lymph and secondary lymphoid organs. We examined the phenotype of endogenous T cells in various non-lymphoid organs and showed that a subset of cells exhibited an apparently naive phenotype and were functionally inactive. FTY720 treatment selectively depleted this population from the non-lymphoid tissues. In addition, RAG-deficient TCR transgenic CD4 and CD8 T cells were present in non-lymphoid tissues in bone marrow chimeric mice and\backslash n in situ\backslash n imaging analysis revealed their location in the parenchymal tissues. Moreover, migration of TCR transgenic T cells to non-lymphoid tissues after adoptive transfer was pertussis-toxin resistant. Overall, the results suggest that naive T cells may circulate through non-lymphoid tissues as part of their normal migratory pathway.","container-title":"European Journal of Immunology","DOI":"10.1002/eji.200535539","ISSN":"0014-2980, 1521-4141","issue":"6","journalAbbreviation":"Eur J Immunol","language":"en","license":"http://onlinelibrary.wiley.com/termsAndConditions\#vor","page":"1423-1433","source":"DOI.org (Crossref)","title":"Evidence that a significant number of naive T cells enter non-lymphoid organs as part of a normal migratory pathway","volume":"36","author":[\{"family":"Cose","given":"Stephen"\},\{"family":"Brammer","given":"Clair"\},\{"family":"Khanna","given":"Kamal M."\},\{"family":"Masopust","given":"David"\},\{"family":"Lefrançois","given":"Leo"\}],"issued":\{"date-parts":[["2006",6]]\}\}\}],"schema":"https://github.com/citation-style-language/schema/raw/master/csl-citation.json"\}$[42–44] | 1.1$\times{10}^{-4}$ | ${10}^{9}$cells/ml |
| $L_{U,0}$ | Initial concentration of unbound IL-6 [45] | 1.1 | pg/ml |
| $L_{B,0}$ | Initial concentration of bound IL-6 | 1.4$\times{10}^{-6}$ | pg/ml |
| $G_{U,0}$ | Initial concentration of unbound GM-CSF [46] | 2.43 | pg/ml |
| $G_{B,0}$ | Initial concentration of bound GM-CSF | 1.6$\times{10}^{-6}$ | pg/ml |
| $C_{U,0}$ | Initial concentration of unbound G-CSF [10] | 0.025 | pg/ml |
| $C_{B,0}$ | Initial concentration of bound G-CSF | 6.5$\times{10}^{-10}$ | pg/ml |
| $F_{U,0}$ | Initial concentration of unbound IFN [47,48] | 0.015 | pg/ml |
| $F_{B,0}$ | Initial concentration of bound IFN | 1.1$\times{10}^{-8}$ | pg/ml |

**Table K. Initial conditions**

| **Variable** | **Description** | **Units** |
| --- | --- | --- |
| $t$ | Time | days |
| $V$ | Viral load | copies/ml |
| $S$ | Number of susceptible cells | ${10}^{9}$cells/ml |
| $I$ | Infected cells | ${10}^{9}$cells/ml |
| $R$ | Resistant cells | ${10}^{9}$cells/ml |
| $M_{\Phi R}$ | Alveolar (resident) macrophages | ${10}^{9}$cells/ml |
| $M_{\Phi I}$ | Inflammatory macrophages | ${10}^{9}$ cells/ml |
| $M$ | Monocytes | ${10}^{9}$ cells/ml |
| $M_{R}$ | Bone marrow reservoir monocytes [9] | ${10}^{9}$ cells/ml |
| $N$ | Neutrophils | ${10}^{9}$ cells/ml |
| $N_{R}$ | Bone marrow reservoir neutrophils [10] | ${10}^{9}$ cells/ml |
| $T$ | CD8+ T cells | ${10}^{9}$ cells/ml |
| $L_{U}$ | Concentration of unbound IL-6 | pg/ml |
| $L_{B}$ | Concentration of bound IL-6 | pg/ml |
| $G_{U}$ | Concentration of unbound GM-CSF | pg/ml |
| $G_{B}$ | Concentration of bound GM-CSF | pg/ml |
| $C_{U}$ | Concentration of unbound G-CSF | pg/ml |
| $C_{B}$ | Concentration of bound G-CSF | pg/ml |
| $C_{BF}$ | Neutrophil G-CSF receptor bound fraction | Unitless |
| $F_{U}$ | Concentration of unbound IFN | pg/ml |
| $F_{B}$ | Concentration of bound IFN | pg/ml |

**Table L. List of variables in the model equations.**

**Description of clinical data**

Blood cells (monocytes, neutrophils, and T cells) and cytokine (type I IFN, IL-6, G-CSF, and GM-CSF) measurements were collected from patients at the Centre hospitalier de l’Université de Montréal (CHUM) and the Jewish General Hospital in Montréal, Canada between March 2020 and August 2020. In this study, we used data from 221 patients: 30 of whom were cancer patients and 24 of whom had immunosuppression (i.e., transplant recipients, patients receiving activated T cell blockers (e.g., teriflunomide) or immunosuppressive drugs, including Solu-Cortef and methylprednisolone. All patients included in our study had primary infections. In addition to the day of symptom onset for each patient, data were collected on days 0,2,7,14, and 30 post-hospitalization. In our simulations, we used the time since symptom onset as the initial time (i.e., t(0)) for each patient. Further information about the clinical study from which the data was collected is in Tremblay et al. [49] and Rébillard et al. [50].

To compare the COVID-19 immunosuppressed VPC with the data, we used the IL-6 concentrations collected on the 11^th^ day post symptom onset from 19 immunosuppressed patients without autoimmune disease [51]. To generate the cancer VPC, we calibrated the initial concentration of neutrophils to a study that included neutropenic patients with COVID-19 [52], as cancer patients tend to have decreased neutrophil counts due to their anti-cancer treatments. Data were available from 23 COVID-19+ neutropenic patients who did not receive exogenous G-CSF therapy, with measurements taken from 14 days before symptom onset to 14 days after symptoms onset. To fit the cancer and reference virtual patient dynamics, CD8+ T cell data were incorporated from 93 COVID-19+ patients with cancer and 1959 COVID-19 patients without cancer [53]. T cells were measured on the 7^th^ and 14^th^ day after symptom onset.

**Testing the inflammation marker accuracy**

We tested the accuracy of the inflammation marker, $\Psi^{j}$ (Eq 2., Main Text), in predicting severity by comparing $\Psi^{j}$ value to the outcome of each patient in our clinical trial data (that means we compared values of $\Psi^{j}$ between severe and moderate patients – for example). As in the inflammation marker for our virtual patients, to calculate $\Psi^{j}$ we used the maximum neutrophil and IL-6 concentrations but substituted the receptor for advanced glycation end products (RAGE), a marker of lung tissue damage [50], because we did not have access to the tissue damage measurements. We quantified the inflammation marker ($\Psi^{j}$) for 40 patients (12 moderate, 12 severe, and 16 critical according to a patient’s required level of respiratory support) [50] and then used a Wilcoxon test to determine whether they were statistically similar. While there was a positive trend in $\Psi^{j}$ value in severe versus moderate patients, we found no statistically significant difference between them (p = 0.13). However, we did find statistically significant differences between the inflammation marker values for patients clinically classified as critical and moderate, and between those classified as critical and severe (S1 Fig), consistent with the results of Jenner et al. [1] and our own.

**High IL-6 concentrations are not necessarily associated with delayed IFN peaks in cancer and immunosuppressed virtual patients**

In Jenner et al. [1], we found a strong relationship between IFN and IL-6, where high maximal IL-6 concentrations were predictive of delayed IFN peak concentrations and thus severity. As expected, this connection was again established in our reference COVID-19 VPC. Interestingly, in the reference VPC, we did not observe any delay in the time to IFN peak concentrations for virtual patients with maximum IL-6 concentrations below 55 pg/ml (S11Ac Fig). However, in both the cancer VPC and the immunosuppressed VPC, we identified several virtual patients (that we later named ‘outlier virtual patients’) with relatively low IL-6 concentrations who experienced delayed IFN peaks (S11Aa and S11Ab Fig).

**Features of outlier virtual patients revealed differences in parameters associated with IFN production**

To better understand the contrasting results of low IL-6 concentrations and IFN peak concentrations, we studied the dynamics and parameter values of selected outlier virtual patients (VP1, VP2, and VP3) in the cancer VPC (S12A Fig). Compared to other virtual patients in the cancer VPC, VP1 had the lowest peak IL-6 concentration, and VP2 and VP3 both had low maximum IL-6 and high maximal CD8+ T cell concentrations (S12A Fig). These three virtual patients differed primarily in their IFN concentrations: VP2 had the highest peak IFN concentration while VP3 had the lowest IFN (S12B Fig), neutrophils (S11Bb Fig), and uninfected cell concentrations (S12C Fig). VP1 had similar neutrophil and uninfected cell dynamics to VP2. Further, VP1 and VP2 had two pronounced IFN peaks, with the first arriving around the second day of infection (S12B Fig), whereas the presence of two peaks was not observed in most other VPs.

By analyzing VP1’s, VP2’s, and VP3’s parameter values, we found that parameters related to IFN production (i.e., $\eta_{F,M_{\varphi I}}$, and $p_{F,I}$) most significantly differed between these three virtual patients (Table M). We next assessed the impact of these two parameter values on predicted immunological dynamics and COVID-19 severity by varying their values. VP3 had a value of $\eta_{F,M_{\varphi I}}$ that was about 3-fold higher than those in VP1 and VP2. Decreasing the value of $\eta_{F,M_{\varphi I}}$ in VP3 resulted in increased IFN concentrations and thus a higher number of uninfected cells (S12C Fig). Similarly, VP2 had a $p_{F,I}$ value around 3 times larger than VP1, and reducing its value caused a decrease in IFN and infected cell concentrations (S12B-12C Fig), see Discussion.

|  | **Parameter** | | | | | | |
| --- | --- | --- | --- | --- | --- | --- | --- |
|  | $\boldsymbol{p}_{\boldsymbol{M}_{\boldsymbol{\varphi I}}\mathbf{,}\boldsymbol{L}}$ | $\boldsymbol{p}_{{\boldsymbol{L}\mathbf{,}\boldsymbol{M}}_{\boldsymbol{\varphi I}}}$ | $\boldsymbol{p}_{\boldsymbol{F}\mathbf{,}\boldsymbol{I}}$ | $\boldsymbol{p}_{\boldsymbol{M}\mathbf{,}\boldsymbol{I}}$ | $\boldsymbol{\eta}_{{\boldsymbol{F}\mathbf{,}\boldsymbol{M}}_{\boldsymbol{\varphi I}}}$ | $\boldsymbol{\epsilon}_{\boldsymbol{F}\mathbf{,}\boldsymbol{I}}$ | $\boldsymbol{p}_{\boldsymbol{F}\mathbf{,}\boldsymbol{M}}$ |
| **VP1** | 2.47 | 1,874 | 0.13 | 0.26 | 1.82$\times$10^-5^ | 1.13$\times$10^-4^ | **3.57** |
| **VP2** | 2.88 | 1,872 | 0.35 | 0.26 | 1.08$\times$10^-5^ | 2.08$\times$10^-4^ | **3.56** |
| **VP3** | 3.05 | 1,872 | 0.33 | 0.22 | 7.30$\times$10^-5^ | 2.17$\times$10^-4^ | **3.58** |

**Table M. Parameter values of ‘outlier virtual patients’.** Shaded values of parameters that differed the most were decreased by 3 times ($p_{F,I}$ in VP3 and VP2) and 7 times ($\eta_{{F,M}_{\varphi I}}$ in VP3) their original values to see the impact of that change on biomarkers dynamics and thus severity.

**SUPPLEMENTARY REFERENCES**

1. Jenner AL, Aogo RA, Alfonso S, Crowe V, Deng X, Smith AP, et al. COVID-19 virtual patient cohort suggests immune mechanisms driving disease outcomes. Yates AJ, editor. PLOS Pathog. 2021;17: e1009753. doi:10.1371/journal.ppat.1009753

2. Norman, P. S. Immunobiology: The immune system in health and disease. Journal of Allergy and Clinical Immunology vol. 96 (Garland Pub., 1995).

3. Kratofil RM, Kubes P, Deniset JF. Monocyte Conversion During Inflammation and Injury. Arterioscler Thromb Vasc Biol. 2017;37: 35–42. doi:10.1161/ATVBAHA.116.308198

4. Lee J, Adler FR, Kim PS. A Mathematical Model for the Macrophage Response to Respiratory Viral Infection in Normal and Asthmatic Conditions. Bull Math Biol. 2017;79: 1979–1998. doi:10.1007/s11538-017-0315-0

5. Jenner AL, Frascoli F, Yun C-O, Kim PS. Optimising Hydrogel Release Profiles for Viro-Immunotherapy Using Oncolytic Adenovirus Expressing IL-12 and GM-CSF with Immature Dendritic Cells. Appl Sci. 2020;10: 2872. doi:10.3390/app10082872

6. Pawelek KA, Dor D, Salmeron C, Handel A. Within-Host Models of High and Low Pathogenic Influenza Virus Infections: The Role of Macrophages. Sun J, editor. PLOS ONE. 2016;11: e0150568. doi:10.1371/journal.pone.0150568

7. Zhang N, Bevan MJ. CD8+ T Cells: Foot Soldiers of the Immune System. Immunity. 2011;35: 161–168. doi:10.1016/j.immuni.2011.07.010

8. De Pillis L, Fister KR, Gu W, Collins C, Daub M, Gross D, et al. Mathematical Model Creation for Cancer Chemo‐Immunotherapy. Comput Math Methods Med. 2009;10: 165–184. doi:10.1080/17486700802216301

9. Cassidy T, Humphries AR, Craig M, Mackey MC. Characterizing Chemotherapy-Induced Neutropenia and Monocytopenia Through Mathematical Modelling. Bull Math Biol. 2020;82: 104. doi:10.1007/s11538-020-00777-0

10. Craig M, Humphries AR, Nekka F, Bélair J, Li J, Mackey MC. Neutrophil dynamics during concurrent chemotherapy and G-CSF administration: Mathematical modelling guides dose optimisation to minimise neutropenia. J Theor Biol. 2015;385: 77–89. doi:10.1016/j.jtbi.2015.08.015

11. Smith P, Wang S, Dowling K, Forsyth K. Leucocyte populations in respiratory syncytial virus‐induced bronchiolitis. J Paediatr Child Health. 2001;37: 146–151. doi:10.1046/j.1440-1754.2001.00618.x

12. Lee HY, Topham DJ, Park SY, Hollenbaugh J, Treanor J, Mosmann TR, et al. Simulation and Prediction of the Adaptive Immune Response to Influenza A Virus Infection. J Virol. 2009;83: 7151–7165. doi:10.1128/JVI.00098-09

13. Klöditz K, Fadeel B. Three cell deaths and a funeral: macrophage clearance of cells undergoing distinct modes of cell death. Cell Death Discov. 2019;5: 65. doi:10.1038/s41420-019-0146-x

14. Zent CS, Elliott MR. Maxed out macs: physiologic cell clearance as a function of macrophage phagocytic capacity. FEBS J. 2017;284: 1021–1039. doi:10.1111/febs.13961

15. Elmore S. Apoptosis: A Review of Programmed Cell Death. Toxicol Pathol. 2007;35: 495–516. doi:10.1080/01926230701320337

16. Tura, S., Cavo, M. & Zinzani, P. L. Hematology. Pathophysiology, Diagnosis and Treatment. (2018).

17. Ginhoux F, Guilliams M. Tissue-Resident Macrophage Ontogeny and Homeostasis. Immunity. 2016;44: 439–449. doi:10.1016/j.immuni.2016.02.024

18. Eftimiea R, Eftimie G. Tumour-Associated Macrophages and Oncolytic Virotherapies: A Mathematical Investigation into a Complex Dynamics. Lett Biomath. 2018;5. doi:10.30707/LiB5.2Eftimiea

19. Patel AA, Zhang Y, Fullerton JN, Boelen L, Rongvaux A, Maini AA, et al. The fate and lifespan of human monocyte subsets in steady state and systemic inflammation. J Exp Med. 2017;214: 1913–1923. doi:10.1084/jem.20170355

20. Craig M, Humphries AR, Mackey MC. An upper bound for the half-removal time of neutrophils from circulation. Blood. 2016;128: 1989–1991. doi:10.1182/blood-2016-07-730325

21. Kim JS, Lee KH, Kim GE, Kim S, Yang JW, Li H, et al. Clinical characteristics and mortality of patients with hematologic malignancies and COVID-19: a systematic review. : 8.

22. Mehra R. Soluble Interleukin 6 Receptor: A Novel Marker of Moderate to Severe Sleep-Related Breathing Disorder. Arch Intern Med. 2006;166: 1725. doi:10.1001/archinte.166.16.1725

23. Penafuerte C, Bautista-Lopez N, Mohamed-Rachid B, Routy J-P, Galipeau J. The Human Ortholog of Granulocyte Macrophage Colony-Stimulating Factor and Interleukin-2 Fusion Protein Induces Potent *Ex vivo* Natural Killer Cell Activation and Maturation. Cancer Res. 2009;69: 9020–9028. doi:10.1158/0008-5472.CAN-09-2322

24. Arnaud P. Les différents interférons : Pharmacologie, mécanismes d’action, tolérance et effets secondaires. Rev Médecine Interne. 2002;23: 449S-458S. doi:10.1016/S0248-8663(02)00659-8

25. Nicola NA, Peterson L, Hilton DJ, Metcalf D. Cellular Processing of Murine Colony-Stimulating Factor (Multi-CSF, GM-CSF, G-CSF) Receptors by Normal Hemopoietic Cells and Cell Lines. Growth Factors. 1988;1: 41–49. doi:10.3109/08977198809000245

26. Mager DE, Jusko WJ. Receptor-Mediated Pharmacokinetic/Pharmacodynamic Model of Interferon-β 1a in Humans. Pharm Res. 2002;19: 1537–1543. doi:10.1023/A:1020468902694

27. Tenhumberg S, Waetzig GH, Chalaris A, Rabe B, Seegert D, Scheller J, et al. Structure-guided Optimization of the Interleukin-6 Trans-signaling Antagonist sgp130. J Biol Chem. 2008;283: 27200–27207. doi:10.1074/jbc.M803694200

28. Snyers L, Fontaine V, Content J. Modulation of Interleukin‐6 Receptors in Human Cells^a^. Ann N Y Acad Sci. 1989;557: 388–395. doi:10.1111/j.1749-6632.1989.tb24031.x

29. Taga T, Kawanishi Y, Hardy RR, Hirano T, Kishimoto T. Receptors for B cell stimulatory factor 2. Quantitation, specificity, distribution, and regulation of their expression. J Exp Med. 1987;166: 967–981. doi:10.1084/jem.166.4.967

30. Yamaguchi M, Michishita M, Hirayoshi K, Yasukawa K, Okuma M, Nagata K. Down-regulation of interleukin 6 receptors of mouse myelomonocytic leukemic cells by leukemia inhibitory factor. J Biol Chem. 1992;267: 22035–22042. doi:10.1016/S0021-9258(18)41631-6

31. Park LS, Friend D, Gillis S, Urdal DL. Characterization of the cell surface receptor for human granulocyte/macrophage colony-stimulating factor. J Exp Med. 1986;164: 251–262. doi:10.1084/jem.164.1.251

32. Barreda D. Regulation of myeloid development and function by colony stimulating factors. Dev Comp Immunol. 2004;28: 509–554. doi:10.1016/j.dci.2003.09.010

33. Branca AA. Interferon receptors. In Vitro Cell Dev Biol. 1988;24: 155–165. doi:10.1007/BF02623541

34. Constantinescu SN, Croze E, Wang C, Murti A, Basu L, Mullersman JE, et al. Role of interferon alpha/beta receptor chain 1 in the structure and transmembrane signaling of the interferon alpha/beta receptor complex. Proc Natl Acad Sci. 1994;91: 9602–9606. doi:10.1073/pnas.91.20.9602

35. Recombinant human interleukin-6 (with HSA). vol. 2020.

36. Razelle, K. Granulocyte-macrophage colony-stimulating factor. vol. 6. Holland-Frei Cancer Medicine; 2003.

37. IFN-beta recombinant protein :: Interferon-beta 1a Recombinant Protein. vol. 2020.

38. Armstrong JD, Gluck EH, Crapo RO, Jones HA, Hughes JM. Lung tissue volume estimated by simultaneous radiographic and helium dilution methods. Thorax. 1982;37: 676–679. doi:10.1136/thx.37.9.676

39. Crapo, J. D., Barry, B. E., Gehr, P., Bachofen, M & Weibel, E. R. Cell number and cell characteristics of the normal human lung. Am Rev Respir Dis. 1982; 126, 332–337.

40. Sharma R, Sharma S. Physiology, Blood Volume. StatPearls. Treasure Island (FL): StatPearls Publishing; 2025. Available: http://www.ncbi.nlm.nih.gov/books/NBK526077/

41. Chaffey N. Alberts, B., Johnson, A., Lewis, J., Raff, M., Roberts, K. and Walter, P. Molecular biology of the cell. 4th edn. Ann Bot. 2003;91: 401–401. doi:10.1093/aob/mcg023

42. Gordon CL, Miron M, Thome JJC, Matsuoka N, Weiner J, Rak MA, et al. Tissue reservoirs of antiviral T cell immunity in persistent human CMV infection. J Exp Med. 2017;214: 651–667. doi:10.1084/jem.20160758

43. Uppal SS, Verma S, Dhot PS. Normal values of CD4 and CD8 lymphocyte subsets in healthy indian adults and the effects of sex, age, ethnicity, and smoking. Cytometry B Clin Cytom. 2003;52B: 32–36. doi:10.1002/cyto.b.10011

44. Cose S, Brammer C, Khanna KM, Masopust D, Lefrançois L. Evidence that a significant number of naive T cells enter non‐lymphoid organs as part of a normal migratory pathway. Eur J Immunol. 2006;36: 1423–1433. doi:10.1002/eji.200535539

45. Kasperska‐Zajac A, Sztylc J, Machura E, Jop G. Plasma IL‐6 concentration correlates with clinical disease activity and serum C‐reactive protein concentration in chronic urticaria patients. Clin Exp Allergy. 2011;41: 1386–1391. doi:10.1111/j.1365-2222.2011.03789.x

46. Lee J, Kim Y, Lim J, Kim M, Han K. G-CSF and GM-CSF Concentrations and Receptor Expression in Peripheral Blood Leukemic Cells from Patients with Chronic Myelogenous Leukemia.

47. Trouillet-Assant S, Viel S, Gaymard A, Pons S, Richard J-C, Perret M, et al. Type I IFN immunoprofiling in COVID-19 patients. J Allergy Clin Immunol. 2020;146: 206-208.e2. doi:10.1016/j.jaci.2020.04.029

48. Rodero MP, Decalf J, Bondet V, Hunt D, Rice GI, Werneke S, et al. Detection of interferon alpha protein reveals differential levels and cellular sources in disease. J Exp Med. 2017;214: 1547–1555. doi:10.1084/jem.20161451

49. Tremblay K, Rousseau S, Zawati MH, Auld D, Chassé M, Coderre D, et al. The Biobanque québécoise de la COVID-19 (BQC19)—A cohort to prospectively study the clinical and biological determinants of COVID-19 clinical trajectories. Lambert JS, editor. PLOS ONE. 2021;16: e0245031. doi:10.1371/journal.pone.0245031

50. Rébillard R-M, Charabati M, Grasmuck C, Filali-Mouhim A, Tastet O, Brassard N, et al. Identification of SARS-CoV-2–specific immune alterations in acutely ill patients. J Clin Invest. 2021;131: e145853. doi:10.1172/JCI145853

51. Monreal E, Sainz de la Maza S, Fernández-Velasco JI, Natera-Villalba E, Rita CG, Rodríguez-Jorge F, et al. The Impact of Immunosuppression and Autoimmune Disease on Severe Outcomes in Patients Hospitalized with COVID-19. J Clin Immunol. 2021;41: 315–323. doi:10.1007/s10875-020-00927-y

52. Lee RJ, Wysocki O, Bhogal T, Shotton R, Tivey A, Angelakas A, et al. Longitudinal characterisation of haematological and biochemical parameters in cancer patients prior to and during COVID-19 reveals features associated with outcome. ESMO Open. 2021;6: 100005. doi:10.1016/j.esmoop.2020.100005

53. Cai G, Gao Y, Zeng S, Yu Y, Liu X, Liu D, et al. Immunological alternation in COVID-19 patients with cancer and its implications on mortality. OncoImmunology. 2021;10: 1854424. doi:10.1080/2162402X.2020.1854424
